# Integrating causal discovery and clinically-relevant insights to explore directional relationships between autistic features, sex at birth, and cognitive abilities

**DOI:** 10.1017/S0033291725000571

**Published:** 2025-03-18

**Authors:** Angela Tseng, Sunday M. Francis, Eric Rawls, Christine Conelea, Nicola M. Grissom, Erich Kummerfeld, Sisi Ma, Suma Jacob

**Affiliations:** 1 Division of Child & Adolescent Psychiatry, Semel Institute of Neuroscience and Human Behavior, University of California Los Angeles, Los Angeles, CA, USA; 2 Department of Psychiatry and Behavioral Sciences, University of Minnesota, Minneapolis, MN, USA; 3 Department of Psychology, University of Minnesota, Minneapolis, MN, USA; 4 Institute for Health Informatics, University of Minnesota, Minneapolis, MN, USA

**Keywords:** Autism, Causal Discovery Analysis, Cognitive, Restricted and Repetitive Behaviors (RRBs), Sex Differences

## Abstract

**Background:**

Access to “big data” is a boon for researchers, fostering collaboration and resource-sharing to accelerate advancements across fields. Yet, disentangling complex datasets has been hindered by methodological limitations, calling for alternative, interdisciplinary approaches to parse manifold multi-directional pathways between clinical features, particularly for highly heterogeneous autism spectrum disorder (ASD). Despite a long history of male-bias in ASD prevalence, no consensus has been reached regarding mechanisms underlying sex-related discrepancies.

**Methods:**

Applying a novel network-theory-based approach, we extracted data-driven, clinically-relevant insights from a well-characterized sample (http://sfari.org/simons-simplex-collection) of autistic males (N = 2175, Age = 8.9 ± 3.5 years) and females (N = 334, Age = 9.2 ± 3.7 years). Expert clinical review of exploratory factor analysis (EFA) results yielded factors of interest in sensory, social, and restricted and repetitive behavior domains. To offset inherent confounds of sample imbalance, we identified a comparison subgroup of males (N = 331) matched to females (by age, IQ). We applied data-driven causal discovery analysis (CDA) using Greedy Fast Causal Inference (GFCI) on three groups (all females, all males, matched males). Structural equation modeling (SEM) extracted measures of model-fit and effect sizes for causal relationships between sex, age-at-enrollment, and IQ on EFA-determined factors.

**Results:**

We identified potential targets for intervention at nodes with mediating or indirect effects. For example, in the female and matched male groups, analyses suggest mitigating RRB domain behaviors may lead to downstream reductions in oppositional and self-injurious behaviors.

**Conclusions:**

Our investigation unveiled sex-specific directional relationships that inform our understanding of differing needs and outcomes associated with biological sex in autism and may serve to further development of targeted interventions.

## Introduction

Autism Spectrum Disorder (ASD) is a complex neurodevelopmental condition characterized by impairments in social communication and interactions (SCI), and restricted, repetitive patterns of behavior, interests, or activities (RRBs) which may include atypical interest in sensory-related (Sens) features of the environment (APA, 2013). While a strong male bias (~4 male:1 female ratio) in ASD prevalence has been reported consistently (Maenner et al., 2023), researchers have yet to reach consensus on the mechanisms and clinical features that underlie these sex (at-birth) discrepancies (Halladay et al., 2015). Moreover, recent estimates suggest that the veracious male-to-female ratio of ASD may be nearer to 2–3:1 (Lai et al., 2015; Posserud et al., 2021), attributed, in part, to under-diagnosis or delayed recognition of autism in girls and women overall (Loomes, Hull, and Mandy, 2017; Whitlock et al., 2020) which can impact their timely access to services and supports (Bargiela, Steward, and Mandy, 2016). Lifespan estimates of 1.8 male:1 female have been reported (Rutherford et al., 2016), reflecting significant differences in the mean age of referral and diagnosis for girls compared to boys, further evidence of delayed recognition of ASD in girls.

Given that diagnostic criteria for ASD have been informed historically by male models of autism, prevailing assessments focusing on paradigmatic autistic features may fail to identify more nuanced female presentations (Stephenson, Norris, and Butter, 2023; Wood-Downie et al., 2021). For example, whereas autistic females are more likely to experience internalizing symptoms (e.g. anxiety, depression), autistic males often exhibit more externalizing behaviors (e.g. hyperactivity, aggression) that prompt earlier clinical evaluations and diagnoses (Lai et al., 2015; Mandy et al., 2011). Sociocultural factors such as higher expectations for females to engage in social communication and interactions may also bias ascertainment of autistic features (Kreiser and White, 2013). Females are more likely to demonstrate ‘camouflaging’ behaviors during human interactions such that they may employ strategies to hide autistic characteristics and fit into ‘neurotypical’ social environments (Ai, Cunningham, and Lai, 2022; Hull et al., 2017b; Livingston, Colvert, Bolton, and Happé, 2019). Although camouflaging may be initially adaptive for social adjustment, later diagnosis, along with the long-term stress of effortful masking and compensation, has been associated with poorer mental health and increased suicidality-risk (Cassidy, Bradley, Shaw, and Baron-Cohen, 2018; Hull, Petrides, and Mandy, 2020). Hence, improving our understanding and recognition of sex-based phenotypic profiles in autism is needed to provide targeted support for males and females during critical early developmental stages (Lai and Szatmari, 2020).

Although male-to-female prevalence ratios may be lower than prior estimations, phenotypically differing sex/gender^1^ profiles in ASD have been observed at an early age. Historically, more intellectual, emotional, and behavioral challenges have been reported in autistic females than males (Duvekot et al., 2017; Frazier, Georgiades, Bishop, and Hardan, 2014; Russell et al., 2022), and these observations have contributed to the specious syllogism that autistic females are more likely to show neurological and functional impairments than males (de Giambattista et al., 2021; Kaat et al., 2020). Yet, an alternate view suggests that females need to demonstrate more severe developmental, behavioral, or intellectual disabilities to garner ASD diagnoses because the female phenotype manifests in a more oblique fashion. For example, children with better communication abilities are diagnosed with autism significantly later than non-verbal and minimally verbal children; girls with complex phrase speech are also diagnosed later than boys with comparable verbal skill levels (Salomone, Charman, McConachie, and Warreyn, 2016), potentially because higher-functioning girls utilize camouflaging strategies to appear less functionally impaired. Consequently, more profound concerns may have to be expressed before females are referred for clinical evaluations.

Although large scale studies have reported a great deal of variation in social communication and cognitive abilities across sexes/genders in ASD (Hull, Mandy, and Petrides, 2017a; Tillmann et al., 2018), observations are persistent of sex-specific presentations of RRBs, a heterogeneous cluster of behavioral symptoms including restricted interests, preoccupation with parts of objects, repetitive motor mannerisms, insistence on sameness, sensory behaviors, and strict adherence to specific routines or rituals. Relative to the well-studied SCI domain, less is known about how RRBs vary according to individual characteristics, including sex and cognitive ability (Frazier, Georgiades, Bishop, and Hardan, 2014; Hartley and Sikora, 2009; Van Wijngaarden-Cremers et al., 2014). Broadly, males have presented with higher RRB-levels (Supekar and Menon, 2015; Szatmari et al., 2012). However, as ASD samples have been predominantly male, a distinct “female” expression (i.e. with less emphasis on RRBs) of the underlying biological liability in autism may be overlooked clinically (Edwards et al., 2024).

Increasing support for discernable sex-based expressions of autistic characteristics has highlighted a need for improved recognition and understanding of mechanisms underlying sex/gender discrepancies in ASD phenotypic profiles. Given the diverse symptomatology, a data-driven approach confers critical insights into the complex interplay between biological, psychological, and environmental mechanisms underlying male and female presentations in autism (Maxwell, Harrison, Rawls, and Zilverstand, 2022). Novel implementations of network theory have shown promise in constructing and analyzing causal or directional relations between symptoms in psychopathology, factoring in strength of interactions as an additional endophenotype (Borsboom, 2017; Borsboom and Cramer, 2013). Accordingly, to investigate the potential of a network approach in ASD, we applied EFA and CDA (see Supplementary Table S3 for terminology) to model the structure and relationships between factors subserving core autism features in a large, well-characterized ASD dataset (Simons Simplex Collection (SSC) v15.3; Fischbach and Lord, 2010; http://sfari.org/simons-simplex-collection). Considering the broad expanse of the autistic symptom space and the relative paucity of research in the RRB and Sens domains (compared to SCIs), we reduced our data dimensionality by integrating clinically-relevant insights into our analyses. Taken together, our approach leverages clinician expertise and data-driven CDA to explore the impact of sex-at-birth and cognitive ability on causal connections in ASD symptom space in a secondary dataset.

## Methods

Our behavioral data was obtained from the Simon Simplex Collection (SSC) v15.3 database; methodology has been described in detail in previously published reports (Fischbach and Lord, 2010) and on the website (http://sfari.org/simons-simplex-collection). Participation in the SSC included diagnostic evaluation, collection of phenotypic measures, and cognitive assessment. Data collection, entry, and validation methods were standardized across collection sites to ensure data reliability. Informed consent was obtained during the original data collection stage and participants opted-in to include their de-identified data in further investigations. Analyses included all participants who completed our measures of interest. Participants with item-level missing data were subsequently excluded from analysis. The total sample consisted of 2509 autistic individuals who were predominantly male (86.7%) and aged 4–18 years old at enrollment. However, because males and females in the main sample were not matched explicitly prior to sample selection, we chose to designate a third subgroup of males drawn from the total sample that were matched pseudo-randomly to females in the cohort by IQ and Age to query whether differences between all male and all female groups would be attributed to IQ, age, or sample size. We employed the R *matchit* function (Ho, Imai, King, and Stuart, 2011) to determine the matched male (MM) subgroup; for each available female participant, the male that matched most closely by four criteria: exact-match to full scale IQ (FSIQ) and nearest-matched to age at enrollment (AGE), verbal IQ (VIQ), and non-verbal IQ (NVIQ). Once the matching criteria were selected, the dataset was locked for analysis. The final three comparison subgroups were as follows: 1) all females (F: N = 334; Age = 9.2 ± 3.7 years), 2) all males (AM: N = 2175; Age = 8.9 ± 3.5 years), and 3) and matched males (MM: N = 331; Age = 8.1 ± 3.2 years). Some individual data were excluded post-selection due to missing item-level responses on assessments, resulting in subgroups that were not equivalent in sample size. We prioritized FSIQ in an effort to obtain males matched to females at similar developmental stages. Multiple variable combinations were gauged for best approach; distance in the matching method was estimated using logistic regression (SM for details). Table 1 depicts participant subgroup characteristics.

**Table 1.** Demographic characteristics of (A) all males (M) and all females (F) in total sample, *p v*alue for M versus F comparison; (B) subset of males matched to females (matched males) and all remaining males (unmatched males). (* indicates significant difference (*p* < 0.05) from all females)

A.				B.	
	Males (M)	Females (F)	*p* value	Matched males (MM)	Unmatched males (UM)
**N (%)**	2175 (86.7%)	334 (13.3%)		331 (15.2%)	1844 (84.8%)
**Age (years)**					
** Mean (SD)**	8.9 (3.5)	9.2 (3.7)	0.206	8.1 (3.2)*	9.1 (3.6)
** Range**	4.0–18.0	4.0–17.9		4.0–17.8	4.0–18.0
**Full scale IQ**					
** Mean (SD)**	82.01 (27.55)	76.29 (27.83)	<.01*	75.65 (27.13)	83.15 (27.48)
** Range**	10–167	18–154		18–141	10–167
**Verbal IQ**					
** Mean (SD)**	78.66 (30.59)	74.92 (31.78)	.039*	58.66 (27.05)*	82.25 (29.80)
** Range**	5–161	9–167		8–143	5–161
**Nonverbal IQ**					
** Mean (SD)**	85.51 (25.82)	79.02 (25.99)	<.01*	87.14 (26.48)*	85.22 (25.69)
** Range**	12–161	22–148		26–148	12–161

### Behavioral measures

We compiled data from individuals from the SSC cohort with available data for IQ measures and responses from all three of the following parent-report assessments of ASD-related features: the Aberrant Behavior Checklist-Community Version (ABC-CV), the Repetitive Behavior Scale-Revised (RBS-R), and the Social Responsiveness Scale (SRS). The ABC-CV (Aman et al., 1995) is an empirically developed, five-factor, 53-item questionnaire that assesses symptoms of irritability and agitation, social withdrawal or lethargy, stereotypic behavior, hyperactivity and non-compliance, and inappropriate speech. The RBS-R (Bodfish et al, 2000; Cuccaro et al, 2007; Mirenda et al, 2010) determines the presence of, characterization, and severity of RRBs; the 43-item Likert-scale questionnaire yields six subscales from a completed form - stereotypy, self-injurious behavior (SIB), compulsive behaviors, ritualistic behaviors, insistence on sameness, and restricted behaviors. Parents also completed the SRS (Constantino and Gruber, 2005), a 65-item, Likert-scale questionnaire, measuring reciprocal social behavior. The completed assessment yields a total score and five subscale scores (social awareness, social cognition, social communication, social motivation, and RRBs). Greater severity of social deficits is demonstrated by higher scores. Each item from each questionnaire was included in our EFA. All included IQ scores were derived from validated and age−/developmentally-appropriate measures; for our analyses, we utilized comparable calculated FSIQ, NVIQ, and VIQ scores. Data collection details for the SSC are available at http://sfari.org/simons-simplex-collection.

### Exploratory factor analysis

Using the ‘psych’ package for R (Revelle, 2017), we reduced data-dimensionality from all included item-level responses (161 items total from ABC-CV, RBSR, and SRS) by applying EFA with maximum likelihood factor extraction and direct oblimin rotation (to allow for correlated factors). Monte Carlo permutation analysis (parallel analysis) was implemented to determine the number of factors to retain (Horn, 1965); using 1000 permutations of the raw data, we retained factors with eigenvalues >95th percentile of permutation eigenvalues (Glorfeld, 2016). EFA yielded 23 factors which were then ordered by variance. If an item was redundant with a higher variance factor, the lower variance factor was eliminated for parsimony. Additionally, only items with loadings = |x| < 0.30 were considered as contributing to the factor (Supplementary Table S1 for a full list), yielding 15 final factors for further analyses (see Table 2). Subsequently, two expert clinicians (S.J., C.C.) reviewed all items within a factor independently and assigned the factors into clinically relevant domains of social communication and interactions (SCI), restricted, repetitive patterns of behavior, interests, or activities (RRBs), and atypical sensory-related interests (Sens). For a few factors (Table 2 and Supplementary Table S1), experts concurred that certain items from the sensory domain overlapped with items from the RRB domain (obsessive-compulsive or self-injurious behaviors), with items from SCI (socioemotional unresponsiveness), or with items from both RRB and SCI (repetitive speech). Consensus reviews were conducted of the resulting factors; inter-rater reliability for category matching was >0.8.

**Table 2.** Exploratory Factor Analysis (EFA) and clinical consensus derived factors of interest

Factor #	Label name	Assessments	Domains
F–01	Oppositional (outburst Behaviors)	ABC	SCI
F–02	Isolated (alone preferred)	ABC;SRS	SCI
F–03	Hand/body movements (recurring mannerisms, stereotypies)	ABC;RBS-R;SRS	RRBs
F–04	Self-injurious behaviors	ABC;RBS-R	RRBs, Sens
F–05	Inflexible (insistent behaviors)	RBS-R;SRS	RRBs
F–06	Social atypicalities (awkward, odd Responses)	SRS	SCI
F–07	Motor overflow (excessive impulsive activity)	ABC;RBS-R	RRBs
F–08	Repetitive speech (perseverative vocal overflow)	ABC;RBS-R;SRS	RRBs, SCI, Sens
F–10	Staring (into space; preoccupied)	ABC;SRS	Sens
F–12	Socioemotional unresponsiveness	ABC;SRS	SCI, Sens
F–14	Body/head ovements (repetitive rocking/turning)	ABC;RBS-R	RRBs
F–15	Socioemotional awareness (responsive/expressive)	SRS	RRBs
F–16	Self-confidence (social communication & interaction)	SRS	SCI
F–17	Sensory and object preoccupation	SRS;RBS-R	Sens
F–23	Obsessive compulsive behaviors	RBS-R	RRBs, Sens

*Assessments:* Aberrant Behavior Checklist-Community Version (ABC-CV); Repetitive Behavior Scale-Revised (RBS-R); Social Responsiveness Scale (SRS).

*Domains:* Social Communication & Interaction (SCI); Restricted, Repetitive Patterns of Behavior, Interests, or Activities (RRBs); Sensory Sensitivity (Sens)

### Causal Discovery Analysis (CDA): Greedy Fast Causal Inference (GFCI)

We used the Greedy Fast Causal Inference (GFCI; freely available Java program Tetrad 6.7.0 (https://github.com/cmu-phil/tetrad) algorithm (Ogarrio, Spirtes, and Ramsey, 2016) to identify the causal structure (qualitative causal relationship, e.g. A is a direct cause of B) that best fits the data. GFCI consists of a two-step process that searches the space of possible causal structures underlying observed relationships between variables. In addition to determining the set of all probabilistic causal relationships among a set of input variables, GFCI can also detect and depict the potential presence of underlying (unmeasured) confounding factors that may explain putatively causal relationships in complex data. In the first step, GFCI searches potential causal models, applying a fast score-based algorithm (Fast Greedy Equivalence Search; FGES) to assign a likelihood score (Bayesian Information Criterion; BIC) that penalizes overly complex models (Chickering, 2002; Ramsey, 2015). During the FGES phase, GFCI greedily adds then removes edges to maximize the model fit. Second, GFCI performs a series of conditional independence tests to rule out preliminary causal relationships not borne out by the data. Prior to analysis, we restricted our model by removing impossible causal relationships. Six relationships were removed - age causing sex, age causing IQ, sex causing age, sex causing IQ, IQ causing sex, and IQ causing age. GFCI parameters were set using a “penalty discount” of 1 to compute penalized likelihoods in the first step (corresponding to the standard Bayesian Information Criteria; BIC) (Schwarz, 1978) and a Fisher *Z p*-value of 0.01 to conduct conditional independence tests in the second step. These are the default settings for these parameters in Tetrad and are typical parameters for applied data analysis.

Using a combination of goodness of fit statistics (BIC) and conditional independence tests (Fisher’s Z), GFCI analyses identify the best fitting models of a causal process, including the possibility of latent common causes (Spirtes, Glymour, and Scheines, 2000), and represent these output visually as Partial Ancestral Graphs (PAGs). In PAGs, variables are represented as nodes, while the type and orientation of connections between two nodes specify the nature of modeled causal relationships (Chickering, 2002; Ogarrio, Spirtes, and Ramsey, 2016; Ramsey, 2015; Spirtes, Glymour, and Scheines, 2000).

### Subgroup models

Separately for each subgroup of females (F), matched males (MM), and all males (AM), we built data-driven causal models of relationships between predetermined (age, verbal IQ; VIQ, non-verbal IQ; NVIQ) and discovered factors underlying ASD-associated characteristics. We then compared the causal models generated for each subgroup to examine sex differences in patterns of ASD symptomatology. Separate graphs for each subgroup were qualitatively compared for edges that were present in one model but not in the other, or edges that were present in more than one model but with different orientations. Edge stability and standardized effect sizes were used to quantitatively compare the strength and direction of each causal relationship.

### Effect size estimation and model fit statistics

To recover effect sizes for causal relationships (e.g. the direct effect of A on B is the amount of change in variable B when variable A is changed by 1 unit while other variables are held constant), we built a Structural Equation Model (SEM) of the GFCI results using the ‘lavaan’ package (Rosseel, 2012) for R (Ramsey, 2015). Raw and standardized effect sizes were estimated by fitting a linear SEM to the PAG. Causal relationships detected by GFCI were included as direct paths in the SEM while confounded or otherwise uncertain relationships were included as covariances.

Graph stability was assessed from 1,000 bootstrap samples, applying the same GFCI analysis to each and aggregating the resulting 1,000 graphs into a table summarizing the proportion of all possible relationships (Supplementary Table S2). Edges (i.e. connections between variables) were classified as directed (in either direction), semi-directed (in either direction), undirected, or bidirected. Bootstrap values indicating the proportion of each edge presence in resampling represent stability or consistency of each connection (moderate ≥50%; high ≥75%). The highest frequency edge type by each variable pair was identified by ensemble rule, i.e. by taking the set of output graphs from the set of resampled data sets and letting the graphs vote on the relationship between every pair of variables, with the highest vote winning. (Kummerfeld and Rix, 2019; Soltis and Soltis, 2003; Stevenson, Kummerfeld, and Merrill, 2021). Edges with an absolute estimated effect size of at least 0.1 were retained to direct focus on relationships with meaningful strength without overlooking potential (but weaker) connections (strong: r ≥ 0.50; moderate: r ≥ 0.30) (Anker et al., 2019; Ogarrio, Spirtes, and Ramsey, 2016; Stevenson, Kummerfeld, and Merrill, 2021).

## Results

To investigate the role of sex (at birth) on causal relationships between AGE, NVIQ, and VIQ and EFA-derived factors across the SCI, RRB, and Sens domains, we compared PAGs for Female Only (F), Matched Male (MM), and All Male (AM) subgroups, focusing on common pathways across subgroups. We report bootstrap values (stability/consistency) and standardized effect sizes (ES) for each relationship; due to large sample size and CDA preference for generating sparse models with strong pathways, p values were uniformly <0.001 for all reported edges (See Table 3 for statistics and included figures for visualization of highlighted relationships).

**Table 3.** Proportion of 1,000 bootstrap resample values (Bootstrap) and standardized effect sizes (ES) for each causal relationship within groups

Direction	Node 1	Causal effect:Red = PositiveBlue = Negative	Direction	Node 2	All females		Matched males		All males	
					Bootstrap	ES	Bootstrap	ES	Bootstrap	ES
**↑**	AGE	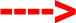	**↓**	Motor overflow (Excessive impulsive activity)	0.90	−0.39	0.58	−0.22	0.38	−0.27
**↑**	AGE	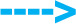	**↑**	Social atypicalities (awkward, odd responses)	0.89	0.37	0.79	0.28		
**↑**	AGE	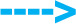	**↑**	Socioemotional unresponsiveness	0.68	0.22				
**↑**	Body/head movements (repetitive rocking/turning)	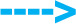	**↑**	Inflexible (insistent behaviors)	0.40	0.25				
**↑**	Body/head movements (repetitive rocking/turning)	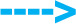	**↑**	Motor overflow (excessive impulsive activity)	0.46	0.26	0.35	0.29	0.56	0.17
**↑**	Body/head movements (repetitive rocking/turning)	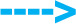	**↑**	Self-injurious behaviors			0.43	0.28		
**↑**	Hand/body movements (recurring mannerisms, stereotypies)	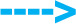	**↑**	Body/head movements (repetitive rocking/turning)	0.71	0.56	0.63	0.52	0.65	0.49
**↑**	Inflexible (insistent behaviors)	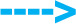	**↑**	Body/head movements (repetitive rocking/turning)			0.16	0.17		
**↑**	Inflexible (insistent behaviors)	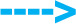	**↑**	Obsessive compulsive behaviors	0.66	0.42	0.64	0.52		
**↑**	Inflexible (insistent behaviors)	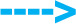	**↑**	Oppositional (outburst behaviors)	0.25	0.21				
**↑**	Inflexible (insistent behaviors)	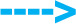	**↑**	Sameness and ritualistic behaviors	0.66	0.42	0.64	0.52		
**↑**	Isolated (alone preferred)	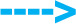	**↑**	Hand/body movements (Recurring mannerisms, stereotypies)	0.32	0.30				
**↑**	Isolated (alone preferred)	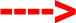	**↓**	Self-confidence	0.57	−0.31				
**↑**	Isolated (alone preferred)	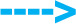	**↑**	Social atypicalities (Awkward, odd responses)			0.47	0.29		
**↑**	Isolated (alone preferred)	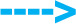	**↑**	Socioemotional unresponsiveness	0.60	0.38	0.72	0.39	0.72	0.31
**↑**	Isolated (alone preferred)	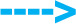	**↑**	Staring (into space; preoccupied)	0.54	0.32	0.62	0.52		
**↑**	Motor overflow (excessive impulsive activity)	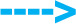	**↑**	Obsessive compulsive behaviors	0.37	0.14				
**↑**	Motor overflow (excessive impulsive activity)	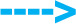	**↑**	oppositional (outburst behaviors)	0.50	0.22	0.58	0.31		
**↑**	NVIQ	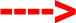	**↓**	Hand/body movements (recurring mannerisms, stereotypies)	0.72	−0.35	0.45	−0.26	0.37	−0.32
**↑**	NVIQ	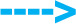	**↑**	Self-confidence	0.49	0.23	0.22	0.28	0.93	0.17
**↑**	NVIQ	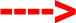	**↓**	Sensory and object preoccupation	0.72	−0.37	0.95	−0.40	0.88	−0.25
**↑**	NVIQ	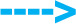	**↑**	socioemotional awareness (responsive/expressive)			0.64	0.33		
**↑**	Obsessive compulsive behaviors	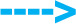	**↑**	Repetitive speech (perseverative vocal overflow)			0.43	0.24		
**↑**	Obsessive compulsive behaviors	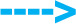	**↑**	Sensory and bbject preoccupation			0.31	0.28		
**↑**	Oppositional (outburst behaviors)	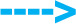	**↑**	Self-injurious behaviors	0.56	0.28	0.37	0.25		
**↑**	Repetitive speech (perseverative vocal overflow)	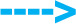	**↑**	hand/ody movements (recurring mannerisms, stereotypies)			0.11	0.21		
**↑**	Self-confidence	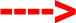	**↓**	Isolated (alone preferred)			0.33	−0.33		
**↑**	Sensory and object preoccupation	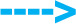	**↑**	Hand/body movements (recurring mannerisms, stereotypies)			0.30	0.26		
**↑**	Social Atypicalities (awkward, odd responses)	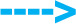	**↑**	Isolated (alone preferred)	0.43	0.33				
**↑**	Social atypicalities (awkward, odd responses)	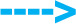	**↑**	Repetitive speech (perseverative vocal overflow)			0.42	0.22		
**↑**	Socioemotional awareness (responsive/expressive)	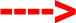	**↓**	Social atypicalities (awkward, odd responses)	0.75	−0.34	0.34	−0.26	0.47	−0.27
**↑**	Socioemotional unresponsiveness	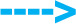	**↑**	Inflexible (insistent behaviors)	0.37	0.22	0.53	0.31		
**↑**	VIQ	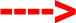	**↓**	repetitive speech (perseverative vocal overflow)	0.61	−0.28				
**↑**	VIQ	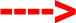	**↓**	Self-injurious behaviors	0.35	−0.20				
**↑**	VIQ	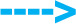	**↑**	Socioemotional awareness (responsive/expressive)	0.53	0.28				

### Females and all males (common paths)

**Figure 1.**
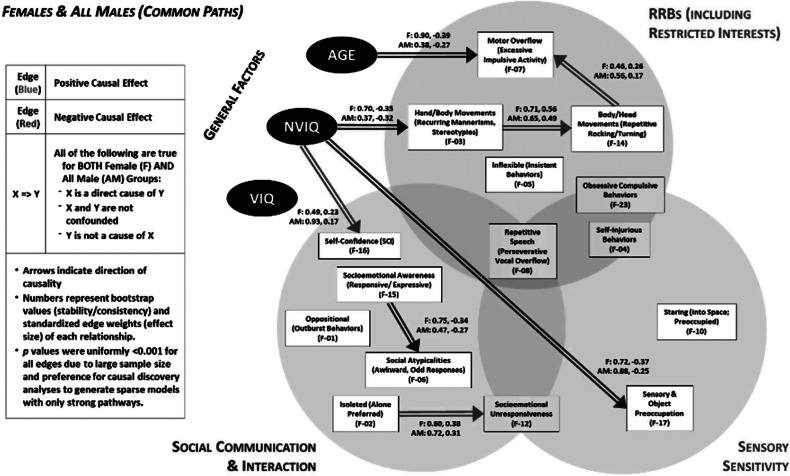
Directed acyclic graph suggested by the Greedy Fast Causal Inference (GFCI) causal discovery algorithm. Double arrows depict causal relations between factors that were common to both female and all male groups.

Several pathways were consistent (presence and direction) across females and males (Figure 1): aligning with clinical observations of development, increasing AGE related to a decrease in motor overflow (Excessive Impulsive Activity), further, higher NVIQ associated with reduced hand/body movements (recurring mannerisms, stereotypies), less sensory and object preoccupation, greater self-confidence, Not surprisingly, being more Isolated (alone preferred) related to increased socioemotional unresponsiveness, and increased socioemotional awareness (responsive/expressive) related to a decrease in social Atypicalities (Awkward, Odd Responses). Suggestive of convergent mechanistic relationships with neural systems underlying movement output and control, we also found associations between motor-related constructs such more hand/body movements (recurring mannerisms, stereotypies associated with an increase in body/head Movements (repetitive rocking/turning) which was, in turn, linked to greater motor overflow (excessive impulsive activity).

### Females and matched males (common paths)

**Figure 2.**
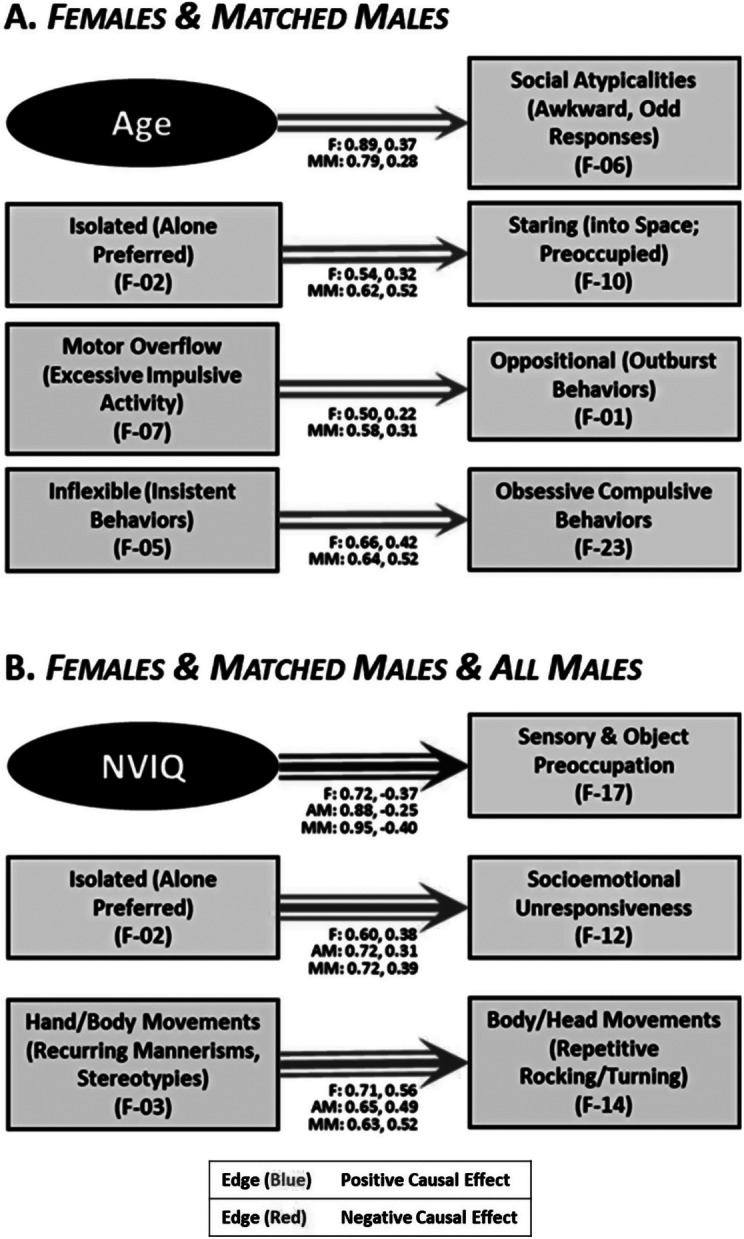
Causal connections between variables present in (A) female and matched male groups (double arrows) and (B) females, matched males, and all male groups (triple arrows).

Common pathways (e.g. Figure 2A) were found between the F and MM subgroups (but not the all male group) such that increased AGE related to an increase in social atypicalities (awkward, odd responses). Increasing motor overflow (excessive impulsive activity) was related to more oppositional (outburst behaviors) activity, which was associated downstream with an increase in self-injurious behaviors. Variables related to social behavior are both linked together and with compulsivity. An increase in socioemotional unresponsiveness related to an increase in inflexible (insistent behaviors), which is associated with an increase in sameness and ritualistic behaviors. Additionally, increased isolation (alone preferred) related to increased staring (into space; preoccupied). For comparative purposes, Figure 2B depicts the relationships that were present in all 3 subgroups (F, AM, MM): increased NVIQ associated with reduced sensory and object preoccupation, being more isolated (alone preferred) related to increased socioemotional unresponsiveness, and more hand/body movements (recurring mannerisms, stereotypies associated with an increase in body/head movements (repetitive rocking/turning).

### Females

**Figure 3.**
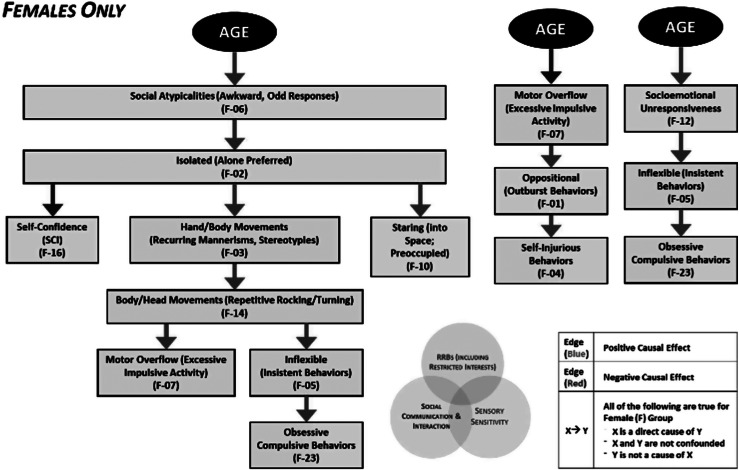
Causal pathways in females identified by CDA originating from AGE.

For females (but not males) in our dataset, CDA yielded multiple directed relationships connecting general factors of AGE (Figure 3), VIQ, and clinical features (EFA-derived factors). Autistic females in our sample showed higher levels of socioemotional unresponsiveness with increasing AGE; this finding aligns with the assertion that females have been historically under−/mis-diagnosed and thus clinically underserved through development. Further, increasing AGE is associated with higher levels of key SCI factors including social atypicalities (awkward, odd responses) as well as socioemotional unresponsiveness, which show direct causal links to nodes in separate SCI, RRB, and Sens domains (See Figure 3).

Additionally, lower VIQ was linked to higher levels of repetitive speech (perseverative vocal overflow) (Figure 5), increased self-injurious behaviors, and reduced socioemotional awareness (Responsive/Expressive). Interestingly, greater motor overflow (Excessive Impulsive Activity) was linked to an increase in obsessive compulsive behaviors, while increased body/head movements (repetitive rocking/turning) was associated with more inflexible (insistent behaviors) which is related to an increase in oppositional (outburst behaviors). These relationships raise the possibility that motor systems play a key role in oppositional and compulsive behavioral tendencies in autistic females, rather than these behaviors being driven by an alternate process (e.g. emotion regulation).

### Matched males

In our subset of males matched to females for IQ and AGE, we observed some similar AGE associations wherein increasing AGE is associated with a decrease in motor overflow (excessive impulsive activity) and an increasing in social atypicalities (awkward, odd responses). In contrast to our female group, matched males did not show an association between increased AGE and increased socioemotional unresponsiveness, suggesting putative sex-divergent trajectories in how emotion is expressed in social contexts or in how autistic individuals are perceived to express such emotions (e.g. where socially-determined expectations for emotion expression differ by sex/gender).

Similar to females, the MM group showed that lower NVIQ was related to increased sensory and object preoccupation. Whereas reduced socioemotional awareness (responsive/expressive) is associated with lower NVIQ in matched males, it is related to lower VIQ in females, raising the possibility that language and verbal abilities contribute differently to social functioning in autism based on sex, societal gender role expectations, or a combination of these factors.

Variables related to excess movement were associated with one another in matched males as they were in females. Higher levels of motor overflow (excessive impulsive activity) were associated with an increase in oppositional (outburst behaviors) and an increase in hand/body movements (recurring mannerisms, stereotypies) was linked to an increase in body/head movements (repetitive rocking/turning). Unlike females, the MM subgroup did not show associations between motor-related processes and oppositional and self-injurious behaviors.

Similar to the female group, in MM, a greater preference for being isolated (alone preferred) was accompanied by more staring (into space; preoccupied) and increased socioemotional unresponsiveness. An increase in socioemotional unresponsiveness was further related to an increase in being inflexible (insistent behaviors), and an increase in being inflexible (insistent behaviors) led to an increase in obsessive compulsive behaviors. Interestingly, MM showed a causal pathway, suggesting that increased self-confidence is associated with a greater preference for being isolated. In contrast, females showed that a greater preference for isolation related to a decrease in self-confidence. This nuanced distinction in ASD phenomenology suggests that gender or sex-related differences may shape how social experiences contribute to an autistic person’s sense of self.

## Discussion

For our investigations, we first used EFA to summarize clinical features across multiple validated assessments and then implemented causal discovery analyses (CDA) to model the structure and relationships between factors subserving general autism features (RRBs, SCI, Sens). CDA is a particularly powerful, data-driven tool that can detect directional influences between factors of interest while considering latent variables and their potentially confounding effects in the greater network. Our network theory-based approach discerned sex-biased, directional relationships between AGE, IQ, and clinical features, demonstrating the potential use of CDA to unearth phenotype-based subtypes that will provide insights into the etiology of ASD and inform therapeutic targeting.

In order to examine how cognitive ability may impact symptom presentation, we considered the influence of verbal and non-verbal IQ in our sex/gender models of ASD. Prior research has suggested that cognitive profiles are affected by an individual’s pattern of performance in verbal and nonverbal reasoning, such that an IQ “discrepancy” or “split,” (e.g. NVIQ>VIQ or VIQ > NVIQ) may serve as a potential autism-related phenotype (Black, Wallace, Sokoloff, and Kenworthy, 2009; Chapman et al., 2010); sex- and age-based differences in cognitive discrepancy profiles have also been reported in ASD (Ankenman et al., 2014; Johnson et al., 2021). In an effort to control for IQ discrepancies that often occur when comparing autistic males and females, we included a subgroup of male participants (MM) matched individually to the smaller female sample by IQ (prioritized) and AGE. Matching procedures yielded a slightly younger male subgroup (MM: Age = 8.1 ± 3.2 years; FSIQ = 75.65 ± 27.13) than female group (F: Age = 9.2 ± 3.7 years; FSIQ = 76.29 ± 27.83); this may be attributed to the lower FSIQ (*p* < .01) overall in the female group than the larger male group (AM: Age = 8.9 ± 3.5 years; FSIQ = 82.01 ± 27.55). As such, younger males were algorithmically selected to better match for FSIQ scores. Additionally, our sampling indicates that males may show a larger NVIQ > VIQ split while female NVIQ and VIQ scores are more concordant.

**Figure 4.**
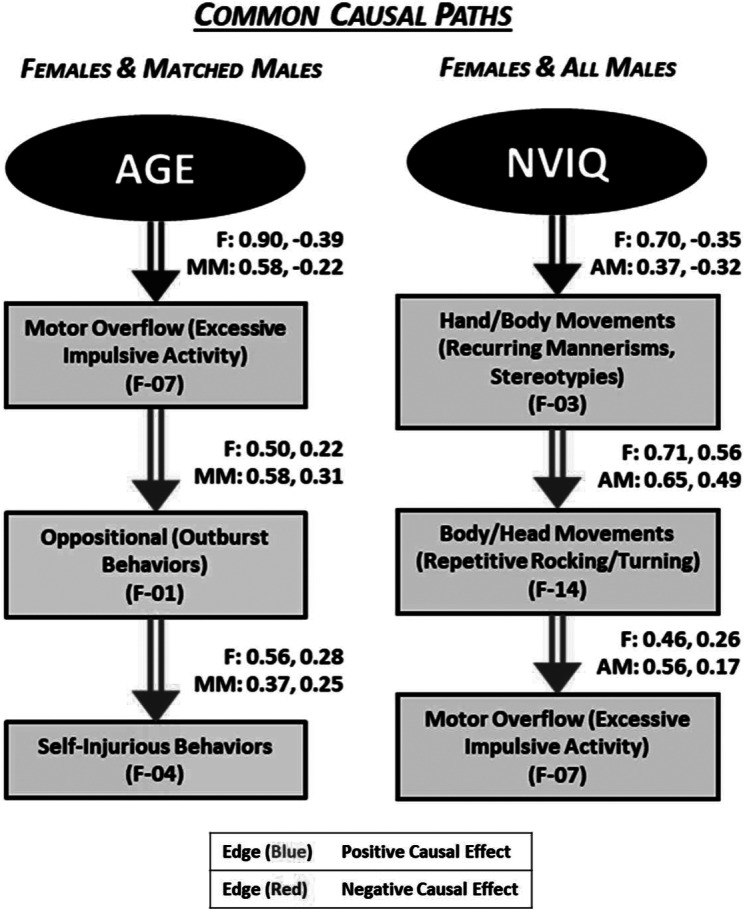
Common causal relationships originating from AGE for females and matched males (double arrows) and from NVIQ for females and all males (double arrows).

Notably, while both AGE and NVIQ were found to be causal ancestors of key factors in all three subgroups (F, AM, MM), VIQ was only an upstream factor for causal paths in the female group. In females, higher VIQ was related to less repetitive speech (perseverative vocal overflow) and more socioemotional awareness (responsive/expressive). For all males and females, NVIQ was directly linked to factors in each domain, such that higher NVIQ associated with more self-confidence (SCI), reduced hand/body movements (recurring mannerisms, stereotypies) (RRBs), and less sensory and object preoccupation (Sens) as noted in Figure 1. The presence of these common pathways suggests a more sex-independent role for NVIQ than VIQ in ASD. Further, while AGE showed a causal influence on both sexes (Figure 1), the variable originated more paths in females, suggesting more age-dependent related presentations and a more dynamic developmental pathway in female adolescence (Figure 4).

**Figure 5.**
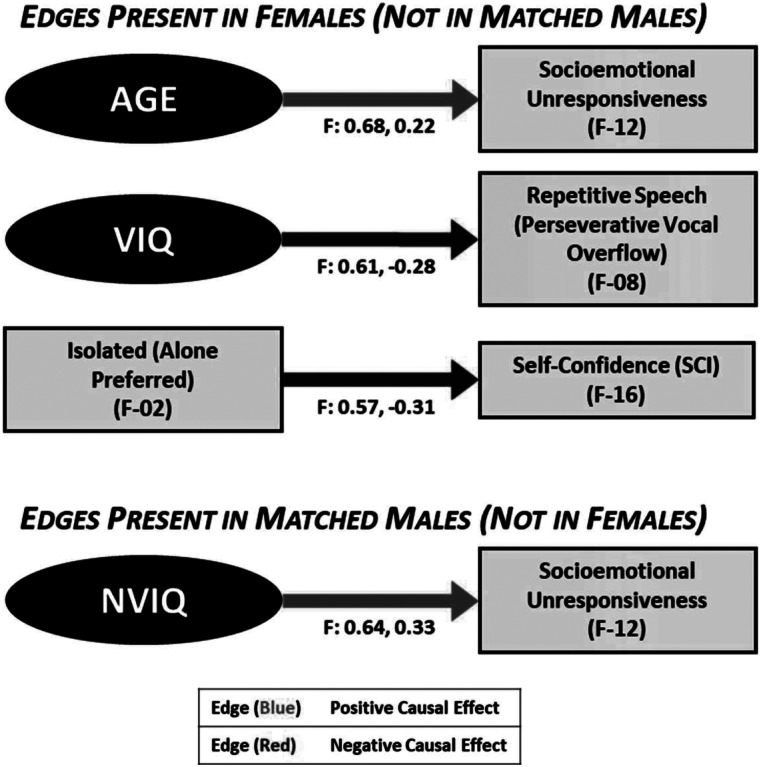
Causal connections present in females (but not matched Males) and in matched males (but not females).

The CDA results and directional relationships align with our ‘real-world’ observations of how clinical presentations vary with IQ and change over time with autistic children (Hull, Mandy, and Petrides, 2017a; Lai and Szatmari, 2020). Differing and distinct relationships were found in subgroups: in our female sample (but not MM), increasing AGE was directly related to increased socioemotional unresponsiveness; in our matched male group (but not F) group, we found that higher NVIQ was directly related to more socioemotional unresponsiveness (Figure 5). We consider that these converging pathways highlight sex/gender differences in sociocultural expectations and phenotypic outcomes. For example, during the critical transitional periods of adolescence and adulthood, youth are exposed to more varied social situations that require complex interpersonal navigation. Females, who had been more successful at camouflaging with peers, may then struggle adjusting to the nuanced social requirements associated with increasing age. In contrast, societal expectations may penalize social disengagement less in males than in females, resulting in more stable observations of responsiveness with age. The association of higher NVIQ with increased socioemotional unresponsiveness in the matched male group may reflect the greater NVIQ>VIQ discrepancy in our male sample; verbal ability is inexorably linked to social communication and would be impacted by the lower VIQ accompanied by higher NVIQ.

Investigating causal pathways between the EFA-derived factors yielded patterns that may enable us to better conceptualize heterogeneity in autism symptom expression across sexes. Further, the mapping of causal influences on ASD-related outcomes may identify targets for intervention at nodes with mediating or indirect causal effects in the pathways. For example, in both our female and matched male groups, we found that an increase in self-injurious behaviors was indirectly caused by an increase in motor overflow (excessive impulsive activity) mediated through an increase in oppositional (outburst behaviors). Our analysis indicates that mitigating behaviors in the RRB domain may lead to a reduction in both oppositional and self-injurious behaviors.

### Limitations

To our knowledge, our analyses represent the first implementation of this causal discovery approach in a large sample of well-characterized youth with ASD. However, we must address limitations in our methodology and dataset. First, given the dynamic nature of the autism field of study, wherein diagnostic criteria, standards, and biases are subject to scrutiny and modification, the SSC v15.3 dataset reflects the state of the field during the period of collection. For example, new to the DSM-5 definition of autism was the inclusion of “hyper or hypo reactivity to sensory input or unusual interest in sensory aspects of the environment” as one of the four restricted/repetitive behavior features defined as atypical sensory processing (American Psychiatric Association, 2013). Consequently, earlier assessments may have been lacking in sensory domain items. Relatedly, the available dataset also inexorably reflects the ongoing sex/gender-biased diagnostic discrepancies discussed earlier. Hence, the male to female ratio of our included sample is closer to 6 males:1 female. This underrepresentation is mirrored in the full dataset (individuals that did not meet our criteria included), and the male to female ratio is 6.37 M: 1F, reflecting the potential under-diagnosis of autistic girls and therefore not recruited to contribute to the Simons Simplex Collection dataset. In response to these concerns, and with the goal of better understanding differences and similarities in male versus female representations of ASD, we created a sample of males matched individually to all available females to offset confounds that may be introduced by age and FSIQ variability. Finally, we acknowledge the inherent limitations of parent/caregiver/other-report measures as a subjective source of data. However, given the development age group of interest (Age < 18 years), questionnaire data from validated measures are the most feasible approach to better understand these vulnerable populations.

### Conclusions

To our knowledge, this is the first study to examine sex differences while modeling direct causal pathways between AGE, IQ, and ASD characteristics across RRB, SCI, and Sens domains in a large sample of autistic youth. By implementing an analytical methodology originally designed for causal discovery from observational data broadly, we unveiled sex-specific directional relationships between multiple clinically-relevant variables. Our findings highlight potential applications for CDA as a means to understand mechanisms of ASD symptomatology, including latent influences on phenotypic outcomes. Further research may impact downstream outcomes (e.g. self-injurious or oppositional behaviors) by illuminating upstream targets for intervention in discovered causal pathways.

## Supporting information

Tseng et al. supplementary material 1 Tseng et al. supplementary material

Tseng et al. supplementary material 2 Tseng et al. supplementary material

Tseng et al. supplementary material 3 Tseng et al. supplementary material

## Footnotes

1 We use the term sex/gender to acknowledge the overlap between biological characteristics (sex) and socially constructed attributes and expectations (gender) as proposed in Springer, Mager Stellman, and Jordan-Young (2012).

## Supplementary material

The supplementary material for this article can be found at http://doi.org/10.1017/S0033291725000571.
