# Supplementary material for: Integrating causal discovery and clinically-relevant insights to explore directional relationships between autistic features, sex at birth, and cognitive abilities
Source: Psychol Med. 2025 Mar 18;55:e89. doi: 10.1017/S0033291725000571 (PMC12080652; doi:10.1017/S0033291725000571)
Supplement: Tseng et al. supplementary material 1 — Tseng et al. supplementary material [file S0033291725000571sup001.docx]

# **SUPPLEMENTAL MATERIALS**

| **Table S1A.** All Exploratory Factor Analysis (EFA) derived factors. Bolding indicates factors that were excluded from final analyses after consensus review by clinical experts. | | |
| --- | --- | --- |
| **Factor #** | **Label Name** | **Assessments** |
| F-01 | Oppositional (Outburst Behaviors) | ABC |
| F-02 | Isolated (Alone Preferred) | ABC; SRS |
| F-03 | Hand/Body Movements (Recurring Mannerisms, Stereotypies) | ABC; RBS-R; SRS |
| F-04 | Self-Injurious Behaviors | ABC; RBS-R |
| F-05 | Inflexible (Insistent Behaviors) | RBS-R; SRS |
| F-06 | Social Atypicalities (Awkward, Odd Responses) | SRS |
| F-07 | Motor Overflow (Excessive Impulsive Activity) | ABC; RBS-R |
| F-08 | Repetitive Speech (Perseverative Vocal Overflow) | ABC; RBS-R; SRS |
| **F-09** | **Decreased Conversational Comprehension** | **SRS** |
| F-10 | Staring (into Space; Preoccupied) | ABC; SRS |
| **F-11** | **Tantrums** | **ABC** |
| F-12 | Socioemotional Unresponsiveness | ABC; SRS |
| **F-13** | **Inflexibility Associated with Social Stress** | **SRS** |
| F-14 | Body/Head Movements (Repetitive Rocking/Turning) | ABC; RBS-R |
| F-15 | Socioemotional Awareness (Responsive/Expressive) | SRS |
| F-16 | Self-Confidence (Social Communication & Interaction) | SRS |
| F-17 | Sensory & Object Preoccupation | RBS-R; SRS |
| **F-18** | **Restlessness** | **ABC** |
| **F-19** | **Temperamental/Irritable** | **ABC** |
| **F-20** | **Restricted Interests/Discussion Topics** | **RBS-R; SRS** |
| **F-21** | **Self-Injurious Behaviors (only characterized by RBS-R)** | **RBS-R** |
| **F-22** | **Teased** | **SRS** |
| F-23 | Obsessive Compulsive Behaviors | RBS-R |
| **Assessments:** Aberrant Behavior Checklist-Community Version (ABC-CV); Repetitive Behavior Scale-Revised (RBS-R); Social Responsiveness Scale (SRS).  **Domains:** Social Communication & Interaction (SCI); Restricted, Repetitive Patterns of Behavior, Interests, or Activities (RRBs); Sensory Sensitivity (Sens) | | |

| **Table S1B.** Individual assessment Items contributing to each derived factor. | | | | |
| --- | --- | --- | --- | --- |
| **Factor Label** | |  | **Assessment Item** | **Source** |
| **F-01: Oppositional (Outburst Behaviors)** | | | |  |
|  | - Aggressive to other children or adults (verbally or physically) | | | ABC |
|  | - Temper tantrums/outbursts | | | ABC |
|  | - Disobedient; difficult to control | | | ABC |
|  | - Uncooperative | | | ABC |
|  | - Does not pay attention to instructions | | | ABC |
|  | - Demands must be met immediately | | | ABC |
|  | - Deliberately ignores directions | | | ABC |
|  | - Has temper outbursts or tantrums when he/she does not get own way | | | ABC |
|  |  | | |  |
| **F-02: Isolated (Alone Preferred)** | | | |  |
|  | - Seeks isolation from others | | | ABC |
|  | - Withdrawn; prefers solitary activities | | | ABC |
|  | - Isolates himself/herself from other children or adults | | | ABC |
|  | - Prefers to be alone | | | ABC |
|  | - Shows few social reactions to others | | | ABC |
|  | - Would rather be alone than with others | | | SRS |
|  |  | | |  |
| **F-03: Hand/Body Movements (Recurring Mannerisms, Stereotypies)** | | | |  |
|  | - Meaningless, recurring body movements | | | ABC |
|  | - Stereotyped behavior; abnormal, repetitive movements | | | ABC |
|  | - Repetitive hand, body, or head movements | | | ABC |
|  | - Waves or shakes the extremities repeatedly | | | ABC |
|  | - HAND/FINGER (Flaps hands, Wiggles or flicks fingers, Claps hands, Waves or shakes hand or arm) | | | RBS-R |
|  | - Has repetitive, odd behaviors such as hand flapping or rocking | | | SRS |
|  |  | | |  |
| **F-04: Self-Injurious Behaviors** | | | |  |
|  | - Injures self on purpose | | | ABC |
|  | - Deliberately hurts himself/herself | | | ABC |
|  | - Does physical violence to self | | | ABC |
|  | - HITS SELF WITH BODY PART (Hits or slaps head, face, or other body area) | | | RBS-R |
|  | - HITS SELF AGAINST SURFACE OR OBJECT (Hits or bangs head or other body part on table, floor or other surface) | | | RBS-R |
|  | - BITES SELF (Bites hand, wrist, arm, lips or tongue) | | | RBS-R |
|  |  | | |  |
| **F-05: Inflexible (Insistent Behaviors)** | | | |  |
|  | - TRAVEL / TRANSPORTATION (Insists on taking certain routes/paths; Must sit in specific location in vehicles; Insists that certain items be present during travel, e.g., toy or material; Insists on seeing or touching certain things or places during travel such as a sign or store) | | | RBS-R |
|  | - Objects to visiting new places | | | RBS-R |
|  | - Becomes upset if interrupted in what he/she is doing | | | RBS-R |
|  | - Insists on sitting at the same place | | | RBS-R |
|  | - Dislikes changes in appearance or behavior of the people around him/her | | | RBS-R |
|  | - Insists on using a particular door | | | RBS-R |
|  | - Resists changing activities; Difficulty with transitions | | | RBS-R |
|  | - Insists on same routine, household, school or work schedule everyday | | | RBS-R |
|  | - Insists that specific things take place at specific times | | | RBS-R |
|  | - Has more difficulty than other children with changes in his or her routine | | | SRS |
|  |  | | |  |
| **F-06: Social Atypicalities (Awkward, Odd Responses)** | | | |  |
|  | - Behaves in ways that seem strange or bizarre | | | SRS |
|  | - Is awkward in turn-taking interactions with peers (e.g., doesn't seem to understand the give-and-take conversations) | | | SRS |
|  | - Has difficulty making friends, even when trying his or her best | | | SRS |
|  | - Does not join group activities unless told to do so | | | SRS |
|  | - Is regarded by other children as odd or weird | | | SRS |
|  | - Is socially awkward, even when he or she is trying to be polite | | | SRS |
|  | - Has trouble keeping up with the flow of a normal conversation. | | | SRS |
|  | - Has difficulty relating to peers | | | SRS |
|  | - Gets teased a lot | | | SRS |
|  |  | | |  |
| **F-07: Motor Overflow (Excessive Impulsive Activity)** | | | |  |
|  | - Excessively active at home, school, work, or elsewhere | | | ABC |
|  | - Boisterous (inappropriately noisy and rough) | | | ABC |
|  | - Impulsive (acts without thinking) | | | ABC |
|  | - Restless, unable to sit still | | | ABC |
|  | - Constantly runs or jumps around the room | | | ABC |
|  | - Tends to be excessively active | | | ABC |
|  | - LOCOMOTION (Turns in circles, Whirls, Jumps, Bounces) | | | RBS-R |
|  |  | | |  |
| **F-08: Repetitive Speech (perseverative vocal overflow)** | | | |  |
|  | - Repetitive speech | | | ABC |
|  | - Talks to self loudly | | | ABC |
|  | - Repeats a word or phrase over and over | | | ABC |
|  | - COMMUNICATION / SOCIAL INTERACTIONS (Repeats same topic(s) during social interactions; Repetitive questioning; Insists on certain topics of conversation; Insists that others say certain things or respond in certain ways during interactions) | | | RBS-R |
|  | - Thinks or talks about the same thing over and over | | | SRS |
|  |  | | |  |
| **F-10: Staring (into space; preoccupied)** | | | |  |
|  | - Preoccupied; stares into space | | | ABC |
|  | - Stares or gazes off into space | | | SRS |
|  |  | | |  |
| **F-12: Unresponsive Socioemotionally** | | | |  |
|  | - Fixed facial expression; lacks emotional responsiveness | | | ABC |
|  | - Resists any form of physical contact | | | ABC |
|  | - Responds negatively to affection | | | ABC |
|  | - Expressions on his or her face don't match what he or she is saying | | | SRS |
|  | - Avoids people who want to be emotionally close to him or her | | | SRS |
|  | - Has overly serious facial expressions | | | SRS |
|  | - Is emotionally distant, doesn't show his or her feelings | | | SRS |
|  |  | | |  |
| **F-14: Body/Head Movements (Repetitive Rocking/Turning)** | | | |  |
|  | - Moves or rolls head back and forth repetitively | | | ABC |
|  | - Rocks body back and forth repeatedly | | | ABC |
|  | - WHOLE BODY (Body rocking, Body swaying) | | | RBS-R |
|  | - HEAD (Rolls head, Nods head, Turns head) | | | RBS-R |
|  |  | | |  |
| **F-15: Socioemotional (Aware, Responsive, Expressive)** | | | |  |
|  | - Is aware of what others are thinking or feeling | | | SRS |
|  | - Is able to communicate his or her feelings to others | | | SRS |
|  | - Is able to understand the meaning of other people's tone of voice and facial expressions | | | SRS |
|  | - Recognizes when something is unfair | | | SRS |
|  | - Is able to imitate others' actions | | | SRS |
|  | - Offers comfort to others when they are sad | | | SRS |
|  | - Responds appropriately to mood changes in others (e.g., when a friend's or playmate's mood changes from happy to sad) | | | SRS |
|  | - Focuses his or her attention to there others are looking or listening | | | SRS |
|  | - Has a sense of humor, understands jokes | | | SRS |
|  | - Knows when he or she is talking too loud or making too much noise | | | SRS |
|  | - Knows when he or she is too close to someone or is invading someone's space | | | SRS |
|  |  | | |  |
| **F-16: Self-confidence** | | | |  |
|  | - Seems self-confident when interacting with others | | | SRS |
|  | - Has good self-confidence | | | SRS |
|  |  | | |  |
| **F-17: Sensory & Object Preoccupation** | | | |  |
|  | - OBJECT USAGE (Spins or twirls objects, Twiddles or slaps or throws objects, Lets objects fall out of hands) | | | RBS-R |
|  | - Preoccupation with part(s) of object rather than the whole object (e.g., buttons on clothes, wheels on toy cars) | | | RBS-R |
|  | - Fascination, preoccupation with movement / things that move (e.g., fans, clocks) | | | RBS-R |
|  | - Shows unusual sensory interests (e.g., mouthing or spinning objects) or strange ways of playing with toys | | | SRS |
|  |  | | |  |
| **F-23 Obsessive Compulsive Behaviors** | | | |  |
|  | - ARRANGING / ORDERING (Arranges certain objects in a particular pattern or place; Need for things to be even or symmetrical) | | | RBS-R |
|  | - COMPLETENESS (Must have doors opened or closed; Takes all items out of a container or area) | | | RBS-R |
|  | - WASHING / CLEANING (Excessively cleans certain body parts; Picks at lint or loose threads) | | | RBS-R |
|  | - CHECKING (Repeatedly checks doors, windows, drawers, appliances, clocks, locks, etc.) | | | RBS-R |
|  | - COUNTING (Counts items or objects; Counts to a certain number or in a certain way) | | | RBS-R |
|  | - HOARDING/SAVING (Collects, hoards or hides specific items) | | | RBS-R |
|  | - REPEATING (Need to repeat routine events; In / out door, up / down from chair, clothing on/off) | | | RBS-R |
|  | - SLEEPING / BEDTIME (Insists on certain pre-bedtime routines; Arranges items in room “just so” prior to bedtime; Insists that certain items be present with him/her during sleep; Insists that another person be present prior to or during sleep) | | | RBS-R |
|  | - SELF-CARE – BATHROOM AND DRESSING (Insists on specific order of activities or tasks related to using the bathroom, to washing, showering, bathing or dressing; Arranges items in a certain way in the bathroom or insists that bathroom items not be moved; Insists on wearing certain clothing items) | | | RBS-R |
|  | - Insists that things remain in the same place(s) (e.g. toys, supplies, furniture, pictures, etc.) | | | RBS-R |
