# Supplementary material for: Integrating causal discovery and clinically-relevant insights to explore directional relationships between autistic features, sex at birth, and cognitive abilities
Source: Psychol Med. 2025 Mar 18;55:e89. doi: 10.1017/S0033291725000571 (PMC12080652; doi:10.1017/S0033291725000571)
Supplement: Tseng et al. supplementary material 2 — Tseng et al. supplementary material [file S0033291725000571sup002.pdf]

Table S2. Causal Discovery Analysis Bootstrap Re-sampling Results for (A.) Females, (B.) All Males, and (C.) Matched Males.

A. FEMALES

	Node 1	Interaction	Node 2	Ensemble	No Edge	-->	<--	-->	<--	-->	<--	o->	<-o	o-o	---
1	AGE	o->	F-07: Motor Overflow (Excessive Impulsive Activity)	0.90	0.00	0.00				0.00		0.90			0.09
2	AGE	o->	F-06: Social Atypicalities (Awkward, Odd Responses)	0.89	0.01	0.01		0.00		0.00		0.89			0.09
3	F-15: Socioemotional Awareness (Responsive/Expressive)	-->	F-06: Social Atypicalities (Awkward, Odd Responses)	0.75	0.03			0.02	0.01	0.75	0.10	0.07			0.02
4	F-17: Sensory & Object Preoccupation	<-o	NVIQ	0.72	0.23		0.03		0.00		0.02		0.72		
5	F-14: Body/Head Movements (Repetitive Rocking/Turning)	<--	F-03: Hand/Body Movements (Recurring Mannerisms, Stereotypies)	0.71	0.00			0.01	0.06	0.21	0.71	0.00	0.00	0.01	
6	F-03: Hand/Body Movements (Recurring Mannerisms, Stereotypies)	<-o	NVIQ	0.70	0.27		0.02		0.01		0.00		0.70		0.00
7	AGE	o->	F-12: Socioemotional Unresponsiveness	0.68	0.25			0.01		0.06		0.68			0.00
8	F-23: Obsessive Compulsive Behaviors	<--	F-05: Inflexible (Insistent Behaviors)	0.66	0.00			0.03	0.03	0.26	0.66	0.00	0.01	0.01	0.00
9	F-08: Repetitive Speech (Perseverative Vocal Overflow)	<-o	VIQ	0.61	0.35		0.01		0.00		0.01		0.61		0.02
10	F-12: Socioemotional Unresponsiveness	<--	F-02: Isolated (Alone Preferred)	0.60	0.00		0.00	0.03	0.03	0.32	0.60		0.01	0.01	0.00
11	F-16: Self-Confidence (SCI)	<--	F-02: Isolated (Alone Preferred)	0.57	0.20			0.01	0.07	0.09	0.57	0.01	0.02	0.00	0.03
12	F-01: Oppositional (Outburst Behaviors)	-->	F-04: Self-Injurious Behaviors	0.56	0.04			0.01	0.02	0.56	0.37	0.00	0.00	0.00	
13	F-10: Staring (into Space; Preoccupied)	<--	F-02: Isolated (Alone Preferred)	0.54	0.09			0.01	0.12	0.15	0.54	0.04	0.02	0.03	0.01
14	F-15: Socioemotional Awareness (Responsive/Expressive)	<-o	VIQ	0.53	0.45		0.01		0.00		0.01		0.53		0.00
15	F-01: Oppositional (Outburst Behaviors)	<--	F-07: Motor Overflow (Excessive Impulsive Activity)	0.50	0.39			0.00	0.06	0.04	0.50				
16	F-16: Self-Confidence (SCI)	<-o	NVIQ	0.49	0.49		0.00						0.49		0.02
17	F-14: Body/Head Movements (Repetitive Rocking/Turning)	-->	F-07: Motor Overflow (Excessive Impulsive Activity)	0.46	0.49			0.02	0.00	0.46	0.03	0.01			
18	F-02: Isolated (Alone Preferred)	<--	F-06: Social Atypicalities (Awkward, Odd Responses)	0.43	0.41			0.02	0.03	0.11	0.43	0.00			0.00
19	F-14: Body/Head Movements (Repetitive Rocking/Turning)	-->	F-05: Inflexible (Insistent Behaviors)	0.40	0.38			0.11	0.03	0.40	0.08	0.00	0.00	0.00	0.00
20	F-01: Oppositional (Outburst Behaviors)	<--	F-05: Inflexible (Insistent Behaviors)	0.37	0.44	0.00		0.01	0.04	0.14	0.37		0.00		
21	F-12: Socioemotional Unresponsiveness	-->	F-05: Inflexible (Insistent Behaviors)	0.37	0.46			0.08	0.01	0.37	0.07	0.00	0.00	0.00	0.00
22	F-07: Motor Overflow (Excessive Impulsive Activity)	<-o	VIQ	0.36	0.64				0.00		0.01		0.36		0.00
23	F-04: Self-Injurious Behaviors	<-o	VIQ	0.35	0.64		0.00		0.00		0.01		0.35		0.00
24	F-02: Isolated (Alone Preferred)	-->	F-03: Hand/Body Movements (Recurring Mannerisms, Stereotypies)	0.32	0.41			0.04	0.04	0.32	0.16	0.01	0.00	0.00	0.01
25	F-15: Socioemotional Awareness (Responsive/Expressive)	<--	F-17: Sensory & Object Preoccupation	0.30	0.53			0.03	0.05	0.08	0.30	0.01	0.00	0.01	
26	F-23: Obsessive Compulsive Behaviors	<--	F-07: Motor Overflow (Excessive Impulsive Activity)	0.25	0.62			0.00	0.07	0.06	0.25				
27	F-17: Sensory & Object Preoccupation	-->	F-23: Obsessive Compulsive Behaviors	0.24	0.50			0.05	0.00	0.24	0.19		0.01		0.00
28	F-10: Staring (into Space; Preoccupied)	<--	F-03: Hand/Body Movements (Recurring Mannerisms, Stereotypies)	0.22	0.57			0.01	0.06	0.12	0.22	0.02	0.01		
29	F-14: Body/Head Movements (Repetitive Rocking/Turning)	<--	F-17: Sensory & Object Preoccupation	0.20	0.60			0.02	0.05	0.13	0.20		0.00	0.00	
30	F-14: Body/Head Movements (Repetitive Rocking/Turning)	-->	F-04: Self-Injurious Behaviors	0.17	0.69			0.08	0.01	0.17	0.05	0.00	0.00	0.00	0.00
31	F-14: Body/Head Movements (Repetitive Rocking/Turning)	-->	F-08: Repetitive Speech (Perseverative Vocal Overflow)	0.17	0.76			0.01	0.01	0.17	0.05	0.00			
32	F-17: Sensory & Object Preoccupation	<-o	VIQ	0.17	0.83				0.00		0.00		0.17		
33	AGE	o->	F-16: Self-Confidence (SCI)	0.17	0.82			0.01		0.01		0.17			0.00
34	AGE	o->	F-04: Self-Injurious Behaviors	0.16	0.82			0.01		0.01		0.16			0.01
35	F-16: Self-Confidence (SCI)	<--	F-08: Repetitive Speech (Perseverative Vocal Overflow)	0.16	0.67			0.00	0.01	0.14	0.16	0.01	0.00		0.01
36	F-07: Motor Overflow (Excessive Impulsive Activity)	-->	F-08: Repetitive Speech (Perseverative Vocal Overflow)	0.16	0.78			0.01		0.16	0.05				
37	F-10: Staring (into Space; Preoccupied)	<--	F-06: Social Atypicalities (Awkward, Odd Responses)	0.15	0.67			0.01	0.05	0.10	0.15	0.01		0.00	
38	F-15: Socioemotional Awareness (Responsive/Expressive)	-->	F-16: Self-Confidence (SCI)	0.15	0.67			0.07	0.00	0.15	0.10	0.00	0.00	0.00	
39	F-12: Socioemotional Unresponsiveness	-->	F-01: Oppositional (Outburst Behaviors)	0.15	0.78			0.03	0.00	0.15	0.04	0.00	0.00		0.00
40	F-08: Repetitive Speech (Perseverative Vocal Overflow)	<-o	NVIQ	0.14	0.84		0.00				0.00		0.14		0.02
41	F-10: Staring (into Space; Preoccupied)	-->	F-08: Repetitive Speech (Perseverative Vocal Overflow)	0.13	0.83			0.01	0.00	0.13	0.03	0.01			
42	F-23: Obsessive Compulsive Behaviors	-->	F-08: Repetitive Speech (Perseverative Vocal Overflow)	0.13	0.76			0.01	0.01	0.13	0.09	0.00			
43	F-17: Sensory & Object Preoccupation	-->	F-03: Hand/Body Movements (Recurring Mannerisms, Stereotypies)	0.12	0.71			0.03	0.02	0.12	0.08	0.01	0.01	0.01	
44	F-12: Socioemotional Unresponsiveness	<-o	VIQ	0.10	0.89								0.10		0.01
45	F-04: Self-Injurious Behaviors	<-o	NVIQ	0.09	0.91								0.09		
46	F-17: Sensory & Object Preoccupation	<--	F-05: Inflexible (Insistent Behaviors)	0.09	0.83			0.03	0.00	0.05	0.09		0.00		
47	F-16: Self-Confidence (SCI)	<-o	VIQ	0.09	0.89		0.01		0.00		0.00		0.09		0.02
48	F-10: Staring (into Space; Preoccupied)	<--	F-14: Body/Head Movements (Repetitive Rocking/Turning)	0.08	0.87			0.00	0.02	0.02	0.08		0.00	0.00	0.00
49	F-03: Hand/Body Movements (Recurring Mannerisms, Stereotypies)	<-o	VIQ	0.08	0.92						0.00		0.08		
50	F-12: Socioemotional Unresponsiveness	-->	F-16: Self-Confidence (SCI)	0.06	0.92			0.01		0.06	0.00				
51	F-01: Oppositional (Outburst Behaviors)	<--	F-06: Social Atypicalities (Awkward, Odd Responses)	0.05	0.92			0.00	0.02	0.01	0.05				
52	F-10: Staring (into Space; Preoccupied)	-->	F-17: Sensory & Object Preoccupation	0.05	0.89			0.01	0.02	0.05	0.02	0.01			0.00
53	F-03: Hand/Body Movements (Recurring Mannerisms, Stereotypies)	-->	F-07: Motor Overflow (Excessive Impulsive Activity)	0.05	0.90			0.01	0.00	0.05	0.04				
54	F-02: Isolated (Alone Preferred)	-->	F-05: Inflexible (Insistent Behaviors)	0.04	0.89			0.03	0.01	0.04	0.02	0.00	0.00	0.00	
55	F-04: Self-Injurious Behaviors	<--	F-08: Repetitive Speech (Perseverative Vocal Overflow)	0.04	0.92			0.00	0.00	0.03	0.04				
56	F-15: Socioemotional Awareness (Responsive/Expressive)	<--	F-07: Motor Overflow (Excessive Impulsive Activity)	0.04	0.94			0.00	0.01	0.00	0.04				
57	F-01: Oppositional (Outburst Behaviors)	<--	F-08: Repetitive Speech (Perseverative Vocal Overflow)	0.04	0.92			0.00	0.00	0.04	0.04				
58	F-03: Hand/Body Movements (Recurring Mannerisms, Stereotypies)	-->	F-08: Repetitive Speech (Perseverative Vocal Overflow)	0.04	0.93			0.02		0.04	0.01	0.00			
59	F-03: Hand/Body Movements (Recurring Mannerisms, Stereotypies)	-->	F-05: Inflexible (Insistent Behaviors)	0.04	0.92			0.02	0.00	0.04	0.02		0.00	0.00	
60	F-23: Obsessive Compulsive Behaviors	<-o	VIQ	0.04	0.96						0.00		0.04		
61	AGE	o->	F-02: Isolated (Alone Preferred)	0.04	0.95	0.00		0.00		0.01		0.04			
62	F-17: Sensory & Object Preoccupation	-->	F-07: Motor Overflow (Excessive Impulsive Activity)	0.04	0.93			0.00		0.04	0.03				
63	F-15: Socioemotional Awareness (Responsive/Expressive)	<--	F-02: Isolated (Alone Preferred)	0.03	0.94			0.01	0.01	0.01	0.03				
64	AGE	o->	F-05: Inflexible (Insistent Behaviors)	0.03	0.97			0.00		0.00		0.03			
65	F-06: Social Atypicalities (Awkward, Odd Responses)	<--	F-08: Repetitive Speech (Perseverative Vocal Overflow)	0.03	0.96			0.00	0.01	0.00	0.03				
66	F-02: Isolated (Alone Preferred)	-->	F-04: Self-Injurious Behaviors	0.03	0.96			0.00		0.03	0.01				
67	F-15: Socioemotional Awareness (Responsive/Expressive)	<-o	NVIQ	0.03	0.97		0.00						0.03		

68	F-02: Isolated (Alone Preferred)	<--	F-07: Motor Overflow (Excessive Impulsive Activity)	0.03	0.97				0.00		0.03				
69	F-17: Sensory & Object Preoccupation	-->	F-04: Self-Injurious Behaviors	0.03	0.95			0.03		0.02	0.00				
70	F-23: Obsessive Compulsive Behaviors	-->	F-02: Isolated (Alone Preferred)	0.03	0.96			0.01	0.01	0.03	0.01	0.00		0.00	
71	F-14: Body/Head Movements (Repetitive Rocking/Turning)	<-o	VIQ	0.03	0.97		0.00						0.03		
72	F-15: Socioemotional Awareness (Responsive/Expressive)	-->	F-03: Hand/Body Movements (Recurring Mannerisms, Stereotypies)	0.03	0.94			0.01	0.01	0.03	0.02				
73	F-15: Socioemotional Awareness (Responsive/Expressive)	<--	F-01: Oppositional (Outburst Behaviors)	0.03	0.95			0.01		0.02	0.03				
74	F-10: Staring (into Space; Preoccupied)	-->	F-01: Oppositional (Outburst Behaviors)	0.03	0.96			0.00	0.00	0.03	0.01	0.00			
75	F-17: Sensory & Object Preoccupation	<--	F-02: Isolated (Alone Preferred)	0.02	0.97			0.00	0.00		0.02		0.00		0.00
76	F-16: Self-Confidence (SCI)	<--	F-07: Motor Overflow (Excessive Impulsive Activity)	0.02	0.98						0.02				
77	F-12: Socioemotional Unresponsiveness	-->	F-23: Obsessive Compulsive Behaviors	0.02	0.96			0.01		0.02	0.01				
78	F-12: Socioemotional Unresponsiveness	<--	F-14: Body/Head Movements (Repetitive Rocking/Turning)	0.02	0.95			0.01	0.01	0.01	0.02	0.00			
79	AGE	o->	F-17: Sensory & Object Preoccupation	0.02	0.98			0.00		0.00		0.02			
80	F-16: Self-Confidence (SCI)	<--	F-06: Social Atypicalities (Awkward, Odd Responses)	0.02	0.97			0.00	0.00	0.00	0.02				
81	F-04: Self-Injurious Behaviors	-->	F-07: Motor Overflow (Excessive Impulsive Activity)	0.02	0.98			0.00		0.02	0.00	0.00			
82	F-07: Motor Overflow (Excessive Impulsive Activity)	<-o	NVIQ	0.02	0.98								0.02		
83	F-05: Inflexible (Insistent Behaviors)	-->	F-08: Repetitive Speech (Perseverative Vocal Overflow)	0.02	0.98			0.00		0.02	0.00				
84	AGE	o->	F-15: Socioemotional Awareness (Responsive/Expressive)	0.01	0.98	0.00				0.00		0.01			0.00
85	F-02: Isolated (Alone Preferred)	-->	F-08: Repetitive Speech (Perseverative Vocal Overflow)	0.01	0.98			0.00		0.01	0.00				0.00
86	AGE	o->	F-14: Body/Head Movements (Repetitive Rocking/Turning)	0.01	0.99							0.01			0.00
87	F-16: Self-Confidence (SCI)	-->	F-04: Self-Injurious Behaviors	0.01	0.99			0.00		0.01					
88	F-10: Staring (into Space; Preoccupied)	<-o	VIQ	0.01	0.99						0.00		0.01		0.00
89	F-12: Socioemotional Unresponsiveness	<--	F-07: Motor Overflow (Excessive Impulsive Activity)	0.01	0.99			0.00	0.00		0.01				
90	F-14: Body/Head Movements (Repetitive Rocking/Turning)	<--	F-06: Social Atypicalities (Awkward, Odd Responses)	0.01	0.98			0.00	0.00	0.00	0.01				
91	F-17: Sensory & Object Preoccupation	-->	F-06: Social Atypicalities (Awkward, Odd Responses)	0.01	0.98			0.00	0.00	0.01	0.01				
92	F-12: Socioemotional Unresponsiveness	<-o	NVIQ	0.01	0.99								0.01		0.00
93	F-03: Hand/Body Movements (Recurring Mannerisms, Stereotypies)	-->	F-04: Self-Injurious Behaviors	0.01	0.99			0.00		0.01					
94	F-14: Body/Head Movements (Repetitive Rocking/Turning)	<--	F-23: Obsessive Compulsive Behaviors	0.01	0.98				0.00	0.01	0.01			0.00	
95	F-14: Body/Head Movements (Repetitive Rocking/Turning)	<-o	NVIQ	0.01	0.99								0.01		
96	F-16: Self-Confidence (SCI)	<--	F-17: Sensory & Object Preoccupation	0.01	0.99				0.00		0.01				
97	F-12: Socioemotional Unresponsiveness	<--	F-15: Socioemotional Awareness (Responsive/Expressive)	0.01	0.98			0.00	0.00	0.01	0.01				
98	F-14: Body/Head Movements (Repetitive Rocking/Turning)	-->	F-02: Isolated (Alone Preferred)	0.01	0.99			0.00	0.00	0.01	0.00				
99	F-17: Sensory & Object Preoccupation	-->	F-08: Repetitive Speech (Perseverative Vocal Overflow)	0.01	0.99			0.00		0.01	0.00				
100	F-01: Oppositional (Outburst Behaviors)	<--	F-02: Isolated (Alone Preferred)	0.01	0.99				0.00	0.00	0.01				
101	F-03: Hand/Body Movements (Recurring Mannerisms, Stereotypies)	-->	F-06: Social Atypicalities (Awkward, Odd Responses)	0.01	0.99					0.01	0.00				
102	F-15: Socioemotional Awareness (Responsive/Expressive)	-->	F-04: Self-Injurious Behaviors	0.01	0.99			0.00		0.01	0.00				
103	AGE	o->	F-08: Repetitive Speech (Perseverative Vocal Overflow)	0.01	0.99					0.00		0.01			
104	F-01: Oppositional (Outburst Behaviors)	-->	F-03: Hand/Body Movements (Recurring Mannerisms, Stereotypies)	0.01	0.99					0.01	0.00				
105	F-04: Self-Injurious Behaviors	<--	F-05: Inflexible (Insistent Behaviors)	0.01	0.99					0.00	0.01				
106	F-23: Obsessive Compulsive Behaviors	<--	F-06: Social Atypicalities (Awkward, Odd Responses)	0.01	0.99				0.01		0.01				
107	F-02: Isolated (Alone Preferred)	<-o	NVIQ	0.01	0.99		0.00						0.01		0.00
108	F-05: Inflexible (Insistent Behaviors)	<-o	NVIQ	0.01	1.00								0.01		
109	F-12: Socioemotional Unresponsiveness	-->	F-17: Sensory & Object Preoccupation	0.01	0.99			0.00	0.00	0.01	0.00				
110	F-16: Self-Confidence (SCI)	<--	F-23: Obsessive Compulsive Behaviors	0.01	0.99				0.00	0.00	0.01				
111	F-12: Socioemotional Unresponsiveness	-->	F-04: Self-Injurious Behaviors	0.00	1.00					0.00					
112	F-12: Socioemotional Unresponsiveness	<--	F-03: Hand/Body Movements (Recurring Mannerisms, Stereotypies)	0.00	0.99			0.00	0.00		0.00		0.00		
113	F-17: Sensory & Object Preoccupation	-->	F-01: Oppositional (Outburst Behaviors)	0.00	1.00					0.00					
114	AGE	o->	F-03: Hand/Body Movements (Recurring Mannerisms, Stereotypies)	0.00	1.00					0.00		0.00			
115	F-10: Staring (into Space; Preoccupied)	<--	F-16: Self-Confidence (SCI)	0.00	1.00						0.00				
116	F-10: Staring (into Space; Preoccupied)	<--	F-23: Obsessive Compulsive Behaviors	0.00	1.00						0.00				
117	AGE	o->	F-01: Oppositional (Outburst Behaviors)	0.00	1.00							0.00			
118	F-01: Oppositional (Outburst Behaviors)	-->	F-23: Obsessive Compulsive Behaviors	0.00	1.00					0.00	0.00				
119	F-01: Oppositional (Outburst Behaviors)	<-o	NVIQ	0.00	1.00								0.00		
120	F-04: Self-Injurious Behaviors	<--	F-06: Social Atypicalities (Awkward, Odd Responses)	0.00	1.00						0.00				
121	F-05: Inflexible (Insistent Behaviors)	<--	F-06: Social Atypicalities (Awkward, Odd Responses)	0.00	1.00						0.00				
122	F-12: Socioemotional Unresponsiveness	-->	F-08: Repetitive Speech (Perseverative Vocal Overflow)	0.00	1.00					0.00	0.00				
123	F-14: Body/Head Movements (Repetitive Rocking/Turning)	-->	F-16: Self-Confidence (SCI)	0.00	1.00			0.00		0.00	0.00				
124	AGE	o->	F-23: Obsessive Compulsive Behaviors	0.00	1.00							0.00			
125	F-01: Oppositional (Outburst Behaviors)	<-o	VIQ	0.00	1.00								0.00		
126	F-06: Social Atypicalities (Awkward, Odd Responses)	-->	F-07: Motor Overflow (Excessive Impulsive Activity)	0.00	1.00					0.00					
127	F-06: Social Atypicalities (Awkward, Odd Responses)	<-o	VIQ	0.00	1.00								0.00		
128	F-10: Staring (into Space; Preoccupied)	-->	F-12: Socioemotional Unresponsiveness	0.00	1.00					0.00					
129	F-10: Staring (into Space; Preoccupied)	-->	F-15: Socioemotional Awareness (Responsive/Expressive)	0.00	1.00					0.00	0.00				
130	F-10: Staring (into Space; Preoccupied)	<--	F-04: Self-Injurious Behaviors	0.00	1.00						0.00				
131	F-10: Staring (into Space; Preoccupied)	<--	F-07: Motor Overflow (Excessive Impulsive Activity)	0.00	1.00						0.00				
132	F-10: Staring (into Space; Preoccupied)	<--	F-05: Inflexible (Insistent Behaviors)	0.00	1.00				0.00						
133	F-14: Body/Head Movements (Repetitive Rocking/Turning)	<--	F-01: Oppositional (Outburst Behaviors)	0.00	1.00						0.00				
134	F-14: Body/Head Movements (Repetitive Rocking/Turning)	<--	F-15: Socioemotional Awareness (Responsive/Expressive)	0.00	1.00				0.00						
135	F-15: Socioemotional Awareness (Responsive/Expressive)	<--	F-08: Repetitive Speech (Perseverative Vocal Overflow)	0.00	1.00						0.00				
136	F-15: Socioemotional Awareness (Responsive/Expressive)	-->	F-23: Obsessive Compulsive Behaviors	0.00	1.00			0.00							
137	F-16: Self-Confidence (SCI)	<--	F-01: Oppositional (Outburst Behaviors)	0.00	1.00						0.00				
138	F-16: Self-Confidence (SCI)	<--	F-03: Hand/Body Movements (Recurring Mannerisms, Stereotypies)	0.00	1.00						0.00				
139	F-16: Self-Confidence (SCI)	<--	F-05: Inflexible (Insistent Behaviors)	0.00	1.00						0.00				
140	F-23: Obsessive Compulsive Behaviors	<--	F-03: Hand/Body Movements (Recurring Mannerisms, Stereotypies)	0.00	1.00						0.00				

B. MATCHED MALES

	Node 1	Interaction	Node 2	Ensemble	No Edge	-->	<--	-->	<--	-->	<--	o->	<-o	o-o	---
1	F-17: Sensory & Object Preoccupation	-->	F-23: Obsessive Compulsive Behaviors	0.97	0.00			0.02	0.00	0.97	0.01		0.00		
2	F-01: Oppositional (Outburst Behaviors)	<--	F-07: Motor Overflow (Excessive Impulsive Activity)	0.90	0.00			0.00	0.08	0.02	0.90				
3	F-23: Obsessive Compulsive Behaviors	-->	F-08: Repetitive Speech (Perseverative Vocal Overflow)	0.89	0.00			0.08	0.00	0.89	0.02	0.00			0.01
4	F-16: Self-Confidence (SCI)	<-o	NVIQ	0.86	0.08		0.03		0.01		0.00		0.86		0.02
5	F-01: Oppositional (Outburst Behaviors)	-->	F-04: Self-Injurious Behaviors	0.81	0.03			0.08	0.05	0.81	0.02	0.00			0.01
6	F-14: Body/Head Movements (Repetitive Rocking/Turning)	-->	F-04: Self-Injurious Behaviors	0.67	0.08	0.00		0.67	0.00	0.22	0.00	0.02			0.00
7	F-12: Socioemotional Unresponsiveness	<--	F-02: Isolated (Alone Preferred)	0.62	0.00		0.00	0.01	0.11	0.09	0.62		0.05		0.12
8	F-12: Socioemotional Unresponsiveness	<-o	VIQ	0.62	0.09		0.01						0.62		0.28
9	F-03: Hand/Body Movements (Recurring Mannerisms, Stereotypies)	-->	F-08: Repetitive Speech (Perseverative Vocal Overflow)	0.59	0.00	0.00		0.59		0.38	0.01	0.01			0.01
10	F-07: Motor Overflow (Excessive Impulsive Activity)	<--	F-08: Repetitive Speech (Perseverative Vocal Overflow)	0.59	0.06			0.01	0.20	0.15	0.59				0.00
11	F-14: Body/Head Movements (Repetitive Rocking/Turning)	<--	F-03: Hand/Body Movements (Recurring Mannerisms, Stereotypies)	0.57	0.00	0.02		0.00	0.57	0.03	0.33	0.01	0.04	0.00	0.01
12	F-04: Self-Injurious Behaviors	<-o	NVIQ	0.55	0.33		0.10		0.01		0.01		0.55		
13	F-14: Body/Head Movements (Repetitive Rocking/Turning)	-->	F-07: Motor Overflow (Excessive Impulsive Activity)	0.54	0.32			0.13	0.00	0.54		0.01			0.00
14	F-14: Body/Head Movements (Repetitive Rocking/Turning)	<--	F-05: Inflexible (Insistent Behaviors)	0.53	0.22	0.00		0.13	0.02	0.05	0.53	0.01	0.00		0.04
15	AGE	<->	F-17: Sensory & Object Preoccupation	0.52	0.00	0.01		0.01		0.06		0.40			0.52
16	F-23: Obsessive Compulsive Behaviors	-->	F-05: Inflexible (Insistent Behaviors)	0.51	0.00			0.44		0.51	0.04	0.00	0.00		0.01
17	F-17: Sensory & Object Preoccupation	<-o	NVIQ	0.50	0.47		0.02		0.00		0.01		0.50		0.01
18	F-03: Hand/Body Movements (Recurring Mannerisms, Stereotypies)	<-o	NVIQ	0.48	0.29		0.20		0.01		0.01		0.48		0.00
19	F-02: Isolated (Alone Preferred)	<--	F-06: Social Atypicalities (Awkward, Odd Responses)	0.47	0.00	0.00		0.12	0.03	0.33	0.47	0.03			0.02
20	F-12: Socioemotional Unresponsiveness	-->	F-16: Self-Confidence (SCI)	0.44	0.45			0.06	0.00	0.44	0.03				0.02
21	F-15: Socioemotional Awareness (Responsive/Expressive)	<-o	NVIQ	0.43	0.54		0.02		0.00		0.01		0.43		0.00
22	F-16: Self-Confidence (SCI)	<--	F-02: Isolated (Alone Preferred)	0.43	0.00		0.01		0.36	0.01	0.43	0.00	0.00		0.19
23	F-01: Oppositional (Outburst Behaviors)	<--	F-05: Inflexible (Insistent Behaviors)	0.42	0.04			0.01	0.42	0.17	0.33		0.00		0.02
24	AGE	o->	F-07: Motor Overflow (Excessive Impulsive Activity)	0.40	0.00	0.01		0.28		0.28		0.40			0.04
25	F-15: Socioemotional Awareness (Responsive/Expressive)	-->	F-06: Social Atypicalities (Awkward, Odd Responses)	0.40	0.00			0.01	0.03	0.40	0.18	0.02		0.00	0.36
26	F-17: Sensory & Object Preoccupation	-->	F-03: Hand/Body Movements (Recurring Mannerisms, Stereotypies)	0.39	0.12		0.00	0.04	0.02	0.39	0.34	0.00	0.04		0.03
27	F-10: Staring (into Space; Preoccupied)	<--	F-08: Repetitive Speech (Perseverative Vocal Overflow)	0.38	0.29	0.01		0.11	0.08	0.06	0.38	0.04	0.00		0.04
28	AGE	o->	F-12: Socioemotional Unresponsiveness	0.38	0.00	0.03		0.08		0.15		0.38			0.37
29	F-10: Staring (into Space; Preoccupied)	-->	F-02: Isolated (Alone Preferred)	0.36	0.00	0.03		0.21	0.05	0.36	0.16	0.04	0.01	0.01	0.14
30	AGE	<->	F-06: Social Atypicalities (Awkward, Odd Responses)	0.35	0.00	0.09		0.09		0.15		0.31			0.35
31	F-06: Social Atypicalities (Awkward, Odd Responses)	<--	F-08: Repetitive Speech (Perseverative Vocal Overflow)	0.34	0.20			0.08	0.35	0.08	0.15				0.16
32	F-12: Socioemotional Unresponsiveness	-->	F-05: Inflexible (Insistent Behaviors)	0.31	0.50			0.09	0.03	0.31	0.02				0.07
33	F-12: Socioemotional Unresponsiveness	-->	F-01: Oppositional (Outburst Behaviors)	0.29	0.41			0.30	0.01	0.24	0.03				0.01
34	F-14: Body/Head Movements (Repetitive Rocking/Turning)	<--	F-17: Sensory & Object Preoccupation	0.29	0.44			0.00	0.29	0.13	0.06	0.02			0.05
35	F-10: Staring (into Space; Preoccupied)	-->	F-12: Socioemotional Unresponsiveness	0.29	0.37	0.02		0.29	0.00	0.16	0.01	0.01			0.14
36	F-10: Staring (into Space; Preoccupied)	<--	F-03: Hand/Body Movements (Recurring Mannerisms, Stereotypies)	0.27	0.35	0.00	0.00	0.02	0.27	0.18	0.12	0.02			0.04
37	F-10: Staring (into Space; Preoccupied)	<--	F-17: Sensory & Object Preoccupation	0.27	0.64			0.02	0.27	0.03	0.03	0.01			0.01
38	F-10: Staring (into Space; Preoccupied)	-->	F-06: Social Atypicalities (Awkward, Odd Responses)	0.27	0.23	0.00		0.27	0.01	0.17	0.03	0.04			0.26
39	F-17: Sensory & Object Preoccupation	<-o	VIQ	0.27	0.53		0.00		0.01		0.06		0.27		0.13
40	F-12: Socioemotional Unresponsiveness	-->	F-14: Body/Head Movements (Repetitive Rocking/Turning)	0.26	0.53		0.00	0.08	0.10	0.26	0.01		0.00		0.01
41	F-15: Socioemotional Awareness (Responsive/Expressive)	<-o	VIQ	0.24	0.52		0.03		0.00		0.05		0.24		0.17
42	F-02: Isolated (Alone Preferred)	-->	F-05: Inflexible (Insistent Behaviors)	0.24	0.42	0.00		0.24	0.09	0.16	0.06	0.01	0.00		0.02
43	F-10: Staring (into Space; Preoccupied)	<--	F-14: Body/Head Movements (Repetitive Rocking/Turning)	0.21	0.59	0.00	0.01	0.04	0.09	0.01	0.22	0.01	0.01		0.02
44	F-02: Isolated (Alone Preferred)	<--	F-03: Hand/Body Movements (Recurring Mannerisms, Stereotypies)	0.21	0.67	0.00		0.02	0.21	0.06	0.02	0.00			0.02
45	F-15: Socioemotional Awareness (Responsive/Expressive)	-->	F-16: Self-Confidence (SCI)	0.20	0.46	0.00		0.11	0.00	0.21	0.14	0.06	0.00		0.02
46	F-04: Self-Injurious Behaviors	<-o	VIQ	0.18	0.72		0.01		0.06		0.03		0.18		
47	F-12: Socioemotional Unresponsiveness	<->	F-15: Socioemotional Awareness (Responsive/Expressive)	0.16	0.50			0.02	0.11	0.07	0.11		0.03		0.16
48	F-03: Hand/Body Movements (Recurring Mannerisms, Stereotypies)	<-o	VIQ	0.16	0.70		0.05		0.02		0.07		0.16		
49	F-14: Body/Head Movements (Repetitive Rocking/Turning)	<-o	NVIQ	0.16	0.82		0.02		0.00		0.00		0.16		0.00
50	F-07: Motor Overflow (Excessive Impulsive Activity)	<-o	NVIQ	0.15	0.83		0.01		0.00		0.00		0.15		0.00
51	F-17: Sensory & Object Preoccupation	-->	F-06: Social Atypicalities (Awkward, Odd Responses)	0.12	0.85			0.12	0.01	0.01	0.01				
52	AGE	<->	F-15: Socioemotional Awareness (Responsive/Expressive)	0.12	0.69	0.08		0.04		0.05		0.03			0.12
53	F-04: Self-Injurious Behaviors	<--	F-05: Inflexible (Insistent Behaviors)	0.12	0.81		0.00	0.00	0.12	0.01	0.04				0.02
54	F-01: Oppositional (Outburst Behaviors)	-->	F-06: Social Atypicalities (Awkward, Odd Responses)	0.11	0.71			0.02	0.09	0.11	0.06				0.01
55	F-03: Hand/Body Movements (Recurring Mannerisms, Stereotypies)	-->	F-07: Motor Overflow (Excessive Impulsive Activity)	0.08	0.87			0.08		0.05	0.00	0.00			
56	F-02: Isolated (Alone Preferred)	<--	F-08: Repetitive Speech (Perseverative Vocal Overflow)	0.08	0.89				0.08	0.01	0.02				0.01
57	F-04: Self-Injurious Behaviors	-->	F-07: Motor Overflow (Excessive Impulsive Activity)	0.07	0.92			0.00	0.01	0.07		0.00			0.00
58	F-15: Socioemotional Awareness (Responsive/Expressive)	<--	F-17: Sensory & Object Preoccupation	0.07	0.88				0.04	0.01	0.07	0.00		0.00	0.00
59	F-03: Hand/Body Movements (Recurring Mannerisms, Stereotypies)	-->	F-04: Self-Injurious Behaviors	0.07	0.92	0.00		0.07		0.00	0.00	0.01	0.00		
60	F-05: Inflexible (Insistent Behaviors)	<-o	VIQ	0.06	0.92		0.00		0.00		0.02		0.06		
61	F-06: Social Atypicalities (Awkward, Odd Responses)	<-o	NVIQ	0.04	0.96						0.00		0.04		
62	F-16: Self-Confidence (SCI)	<--	F-08: Repetitive Speech (Perseverative Vocal Overflow)	0.04	0.93				0.04		0.04				
63	AGE	-->	F-02: Isolated (Alone Preferred)	0.03	0.93	0.00		0.01		0.03		0.02			0.00
64	F-14: Body/Head Movements (Repetitive Rocking/Turning)	<--	F-23: Obsessive Compulsive Behaviors	0.03	0.92	0.00		0.01	0.02	0.02	0.03		0.00		0.00
65	F-14: Body/Head Movements (Repetitive Rocking/Turning)	<->	VIQ	0.03	0.92		0.02		0.00		0.01		0.02		0.03
66	F-17: Sensory & Object Preoccupation	-->	F-04: Self-Injurious Behaviors	0.03	0.97			0.03		0.00					
67	F-05: Inflexible (Insistent Behaviors)	<--	F-06: Social Atypicalities (Awkward, Odd Responses)	0.03	0.95				0.02		0.03				
68	AGE	o->	F-16: Self-Confidence (SCI)	0.02	0.94	0.00		0.02		0.02		0.02			

69	F-08: Repetitive Speech (Perseverative Vocal Overflow)	<-o	VIQ	0.02	0.96		0.00		0.01		0.01		0.02		
70	AGE	o->	F-05: Inflexible (Insistent Behaviors)	0.02	0.96			0.01		0.00		0.02			
71	F-05: Inflexible (Insistent Behaviors)	<--	F-08: Repetitive Speech (Perseverative Vocal Overflow)	0.02	0.97				0.01	0.00	0.02	0.00			
72	F-15: Socioemotional Awareness (Responsive/Expressive)	<--	F-05: Inflexible (Insistent Behaviors)	0.02	0.97			0.00	0.01	0.00	0.02				
73	F-08: Repetitive Speech (Perseverative Vocal Overflow)	<->	NVIQ	0.02	0.97		0.00						0.02		0.02
74	F-23: Obsessive Compulsive Behaviors	-->	F-02: Isolated (Alone Preferred)	0.02	0.95			0.02	0.01	0.01	0.01		0.00		
75	F-12: Socioemotional Unresponsiveness	<-o	NVIQ	0.01	0.98								0.01		0.01
76	F-16: Self-Confidence (SCI)	-->	F-06: Social Atypicalities (Awkward, Odd Responses)	0.01	0.98					0.01	0.00				0.01
77	AGE	o->	F-08: Repetitive Speech (Perseverative Vocal Overflow)	0.01	0.98			0.01		0.00		0.01			0.00
78	F-14: Body/Head Movements (Repetitive Rocking/Turning)	-->	F-02: Isolated (Alone Preferred)	0.01	0.98	0.00		0.01		0.01	0.00				
79	F-16: Self-Confidence (SCI)	<-o	VIQ	0.01	0.98		0.00		0.00		0.00		0.01		0.00
80	AGE	-->	F-01: Oppositional (Outburst Behaviors)	0.01	0.99	0.01		0.01							
81	F-04: Self-Injurious Behaviors	<--	F-08: Repetitive Speech (Perseverative Vocal Overflow)	0.01	0.99				0.01	0.00	0.00				
82	F-02: Isolated (Alone Preferred)	<-o	VIQ	0.01	1.00								0.01		
83	F-10: Staring (into Space; Preoccupied)	<--	F-15: Socioemotional Awareness (Responsive/Expressive)	0.01	0.99				0.01		0.00				
84	F-12: Socioemotional Unresponsiveness	-->	F-23: Obsessive Compulsive Behaviors	0.01	0.99			0.00	0.00	0.01					
85	F-16: Self-Confidence (SCI)	<--	F-07: Motor Overflow (Excessive Impulsive Activity)	0.01	0.99				0.00		0.01				
86	F-15: Socioemotional Awareness (Responsive/Expressive)	-->	F-23: Obsessive Compulsive Behaviors	0.00	1.00				0.00	0.00					
87	F-23: Obsessive Compulsive Behaviors	<-o	VIQ	0.00	1.00						0.00		0.00		
88	F-04: Self-Injurious Behaviors	-->	F-06: Social Atypicalities (Awkward, Odd Responses)	0.00	0.99				0.00	0.00	0.00				
89	F-14: Body/Head Movements (Repetitive Rocking/Turning)	-->	F-15: Socioemotional Awareness (Responsive/Expressive)	0.00	1.00					0.00					
90	F-23: Obsessive Compulsive Behaviors	-->	F-04: Self-Injurious Behaviors	0.00	1.00			0.00		0.00	0.00				
91	AGE	<->	F-10: Staring (into Space; Preoccupied)	0.00	0.99	0.00		0.00		0.00					0.00
92	F-05: Inflexible (Insistent Behaviors)	<-o	NVIQ	0.00	1.00								0.00		
93	F-07: Motor Overflow (Excessive Impulsive Activity)	<-o	VIQ	0.00	1.00								0.00		
94	F-15: Socioemotional Awareness (Responsive/Expressive)	-->	F-07: Motor Overflow (Excessive Impulsive Activity)	0.00	0.99			0.00		0.00	0.00				
95	F-16: Self-Confidence (SCI)	-->	F-01: Oppositional (Outburst Behaviors)	0.00	1.00					0.00					
96	F-17: Sensory & Object Preoccupation	<--	F-02: Isolated (Alone Preferred)	0.00	1.00				0.00						0.00
97	AGE	o->	F-14: Body/Head Movements (Repetitive Rocking/Turning)	0.00	1.00							0.00			
98	AGE	o->	F-03: Hand/Body Movements (Recurring Mannerisms, Stereotypies)	0.00	1.00							0.00			
99	F-01: Oppositional (Outburst Behaviors)	<--	F-02: Isolated (Alone Preferred)	0.00	1.00				0.00				0.00		
100	F-10: Staring (into Space; Preoccupied)	<--	F-01: Oppositional (Outburst Behaviors)	0.00	1.00			0.00	0.00		0.00				
101	F-10: Staring (into Space; Preoccupied)	<--	F-07: Motor Overflow (Excessive Impulsive Activity)	0.00	1.00				0.00						
102	F-10: Staring (into Space; Preoccupied)	<-o	NVIQ	0.00	1.00								0.00		
103	F-15: Socioemotional Awareness (Responsive/Expressive)	-->	F-01: Oppositional (Outburst Behaviors)	0.00	1.00					0.00	0.00				
104	F-15: Socioemotional Awareness (Responsive/Expressive)	-->	F-03: Hand/Body Movements (Recurring Mannerisms, Stereotypies)	0.00	1.00					0.00	0.00				
105	F-16: Self-Confidence (SCI)	-->	F-04: Self-Injurious Behaviors	0.00	1.00					0.00					
106	F-17: Sensory & Object Preoccupation	-->	F-07: Motor Overflow (Excessive Impulsive Activity)	0.00	1.00			0.00							
107	F-17: Sensory & Object Preoccupation	-->	F-08: Repetitive Speech (Perseverative Vocal Overflow)	0.00	1.00			0.00							

C. ALL MALES

	Node 1	Interaction	Node 2	Ensemble	No Edge	-->	<--	-->	<--	-->	<--	o->	<-o	o-o	---
1	F-16: Self-Confidence (SCI)	<-o	NVIQ	0.94	0.03		0.04						0.94		
2	F-01: Oppositional (Outburst Behaviors)	<--	F-07: Motor Overflow (Excessive Impulsive Activity)	0.93	0.00				0.05	0.02	0.93				
3	F-17: Sensory & Object Preoccupation	-->	F-23: Obsessive Compulsive Behaviors	0.92	0.00			0.04		0.92	0.02		0.03		0.00
4	F-17: Sensory & Object Preoccupation	<-o	NVIQ	0.88	0.11		0.02						0.88		
5	F-23: Obsessive Compulsive Behaviors	-->	F-08: Repetitive Speech (Perseverative Vocal Overflow)	0.87	0.00			0.07		0.87	0.04	0.03			0.00
6	F-12: Socioemotional Unresponsiveness	<-o	VIQ	0.81	0.01		0.00				0.00		0.81		0.18
7	F-01: Oppositional (Outburst Behaviors)	-->	F-04: Self-Injurious Behaviors	0.80	0.02			0.06	0.09	0.80	0.02				0.00
8	F-12: Socioemotional Unresponsiveness	<--	F-02: Isolated (Alone Preferred)	0.72	0.00			0.00	0.11	0.03	0.72		0.04		0.10
9	AGE	<->	F-17: Sensory & Object Preoccupation	0.70	0.00	0.02		0.02		0.05		0.21			0.70
10	F-15: Socioemotional Awareness (Responsive/Expressive)	<-o	NVIQ	0.69	0.28		0.02						0.69		
11	F-14: Body/Head Movements (Repetitive Rocking/Turning)	-->	F-04: Self-Injurious Behaviors	0.67	0.05			0.67		0.27	0.00	0.01		0.00	0.00
12	F-07: Motor Overflow (Excessive Impulsive Activity)	<--	F-08: Repetitive Speech (Perseverative Vocal Overflow)	0.66	0.05			0.01	0.16	0.11	0.66				0.01
13	F-14: Body/Head Movements (Repetitive Rocking/Turning)	<--	F-03: Hand/Body Movements (Recurring Mannerisms, Stereotypies)	0.65	0.00	0.01	0.00	0.00	0.65	0.01	0.31	0.00	0.01		0.00
14	F-14: Body/Head Movements (Repetitive Rocking/Turning)	<--	F-05: Inflexible (Insistent Behaviors)	0.65	0.11			0.12	0.03	0.07	0.65	0.00	0.00		0.02
15	F-23: Obsessive Compulsive Behaviors	-->	F-05: Inflexible (Insistent Behaviors)	0.61	0.00			0.33	0.00	0.61	0.03	0.03			0.00
16	F-03: Hand/Body Movements (Recurring Mannerisms, Stereotypies)	-->	F-08: Repetitive Speech (Perseverative Vocal Overflow)	0.61	0.00			0.61		0.38	0.01				0.00
17	AGE	<->	F-12: Socioemotional Unresponsiveness	0.59	0.00	0.03		0.06		0.12		0.20			0.59
18	F-14: Body/Head Movements (Repetitive Rocking/Turning)	-->	F-07: Motor Overflow (Excessive Impulsive Activity)	0.56	0.30			0.13	0.00	0.56		0.01			
19	F-04: Self-Injurious Behaviors	<-o	NVIQ	0.55	0.34		0.11						0.55		
20	F-01: Oppositional (Outburst Behaviors)	<--	F-05: Inflexible (Insistent Behaviors)	0.52	0.02				0.52	0.13	0.31		0.00		0.02
21	F-02: Isolated (Alone Preferred)	<--	F-06: Social Atypicalities (Awkward, Odd Responses)	0.51	0.00	0.00		0.11	0.11	0.24	0.51	0.03	0.00		0.01
22	F-12: Socioemotional Unresponsiveness	-->	F-05: Inflexible (Insistent Behaviors)	0.49	0.25		0.00	0.10	0.02	0.49	0.02				0.13
23	F-16: Self-Confidence (SCI)	<--	F-02: Isolated (Alone Preferred)	0.48	0.00		0.00		0.38	0.01	0.49	0.00	0.00		0.12
24	F-15: Socioemotional Awareness (Responsive/Expressive)	-->	F-06: Social Atypicalities (Awkward, Odd Responses)	0.47	0.00			0.01	0.05	0.47	0.20	0.01			0.26
25	F-17: Sensory & Object Preoccupation	-->	F-03: Hand/Body Movements (Recurring Mannerisms, Stereotypies)	0.46	0.09		0.00	0.03	0.03	0.46	0.34		0.02		0.04
26	AGE	<->	F-06: Social Atypicalities (Awkward, Odd Responses)	0.43	0.00	0.05		0.12		0.22		0.18			0.43
27	AGE	-->	F-07: Motor Overflow (Excessive Impulsive Activity)	0.38	0.00	0.00		0.38		0.35		0.23			0.05
28	F-12: Socioemotional Unresponsiveness	-->	F-01: Oppositional (Outburst Behaviors)	0.37	0.43			0.37	0.01	0.18	0.00				0.01
29	F-12: Socioemotional Unresponsiveness	-->	F-16: Self-Confidence (SCI)	0.37	0.54			0.03	0.00	0.37	0.05				0.01
30	F-03: Hand/Body Movements (Recurring Mannerisms, Stereotypies)	<-o	NVIQ	0.37	0.30		0.34						0.37		

31	F-10: Staring (into Space; Preoccupied)	-->	F-12: Socioemotional Unresponsiveness	0.30	0.41	0.02		0.30	0.01	0.08	0.02	0.01			0.16
32	F-10: Staring (into Space; Preoccupied)	-->	F-02: Isolated (Alone Preferred)	0.30	0.00	0.05		0.21	0.04	0.30	0.12	0.11	0.01	0.00	0.18
33	F-15: Socioemotional Awareness (Responsive/Expressive)	-->	F-16: Self-Confidence (SCI)	0.29	0.11	0.01		0.15	0.01	0.29	0.17	0.20	0.00	0.00	0.07
34	F-10: Staring (into Space; Preoccupied)	<--	F-17: Sensory & Object Preoccupation	0.28	0.58			0.03	0.28	0.04	0.03	0.02			0.02
35	F-02: Isolated (Alone Preferred)	<--	F-03: Hand/Body Movements (Recurring Mannerisms, Stereotypies)	0.27	0.45			0.03	0.28	0.17	0.06				0.02
36	F-14: Body/Head Movements (Repetitive Rocking/Turning)	<--	F-17: Sensory & Object Preoccupation	0.27	0.52			0.00	0.27	0.09	0.04	0.02			0.05
37	F-03: Hand/Body Movements (Recurring Mannerisms, Stereotypies)	<-o	VIQ	0.27	0.67		0.00		0.00		0.05		0.27		0.00
38	F-10: Staring (into Space; Preoccupied)	<--	F-08: Repetitive Speech (Perseverative Vocal Overflow)	0.26	0.37	0.02		0.20	0.03	0.06	0.26	0.05	0.00		0.03
39	F-10: Staring (into Space; Preoccupied)	<->	F-06: Social Atypicalities (Awkward, Odd Responses)	0.25	0.15	0.00		0.24	0.01	0.20	0.10	0.05			0.25
40	F-12: Socioemotional Unresponsiveness	-->	F-14: Body/Head Movements (Repetitive Rocking/Turning)	0.25	0.44			0.21	0.08	0.25	0.01		0.00		0.02
41	F-04: Self-Injurious Behaviors	<-o	VIQ	0.24	0.68		0.01		0.03		0.04		0.24		0.00
42	F-06: Social Atypicalities (Awkward, Odd Responses)	-->	F-08: Repetitive Speech (Perseverative Vocal Overflow)	0.23	0.12			0.23	0.23	0.18	0.10				0.13
43	F-10: Staring (into Space; Preoccupied)	<--	F-03: Hand/Body Movements (Recurring Mannerisms, Stereotypies)	0.23	0.19	0.02		0.07	0.23	0.22	0.20	0.02			0.04
44	F-14: Body/Head Movements (Repetitive Rocking/Turning)	<-o	NVIQ	0.20	0.77		0.03						0.20		
45	AGE	<->	F-15: Socioemotional Awareness (Responsive/Expressive)	0.19	0.58	0.05		0.07		0.08		0.03			0.19
46	F-03: Hand/Body Movements (Recurring Mannerisms, Stereotypies)	-->	F-07: Motor Overflow (Excessive Impulsive Activity)	0.17	0.76			0.17	0.00	0.07		0.00			
47	F-04: Self-Injurious Behaviors	<--	F-05: Inflexible (Insistent Behaviors)	0.16	0.79			0.00	0.16	0.01	0.03		0.00		0.01
48	F-02: Isolated (Alone Preferred)	-->	F-05: Inflexible (Insistent Behaviors)	0.16	0.69	0.00		0.16	0.04	0.06	0.03	0.00			0.01
49	F-10: Staring (into Space; Preoccupied)	<--	F-14: Body/Head Movements (Repetitive Rocking/Turning)	0.14	0.73	0.00	0.00	0.06	0.03	0.01	0.14	0.01	0.01		0.01
50	F-17: Sensory & Object Preoccupation	-->	F-06: Social Atypicalities (Awkward, Odd Responses)	0.14	0.79			0.14	0.04	0.01	0.02				
51	F-12: Socioemotional Unresponsiveness	<--	F-15: Socioemotional Awareness (Responsive/Expressive)	0.13	0.60			0.02	0.13	0.05	0.09		0.02		0.09
52	F-15: Socioemotional Awareness (Responsive/Expressive)	<-o	VIQ	0.13	0.74		0.00		0.00		0.01		0.13		0.12
53	F-07: Motor Overflow (Excessive Impulsive Activity)	<-o	NVIQ	0.13	0.85		0.03						0.13		
54	F-01: Oppositional (Outburst Behaviors)	<--	F-06: Social Atypicalities (Awkward, Odd Responses)	0.11	0.77			0.01	0.12	0.07	0.03				0.01
55	F-04: Self-Injurious Behaviors	-->	F-07: Motor Overflow (Excessive Impulsive Activity)	0.11	0.87			0.00	0.01	0.11	0.00				
56	F-05: Inflexible (Insistent Behaviors)	<-o	VIQ	0.09	0.82		0.00		0.01		0.02		0.09		0.00
57	F-06: Social Atypicalities (Awkward, Odd Responses)	<-o	NVIQ	0.08	0.92		0.00						0.08		
58	F-17: Sensory & Object Preoccupation	<->	VIQ	0.07	0.88		0.00				0.00		0.04		0.07
59	F-05: Inflexible (Insistent Behaviors)	<--	F-06: Social Atypicalities (Awkward, Odd Responses)	0.07	0.90				0.07		0.04				
60	F-03: Hand/Body Movements (Recurring Mannerisms, Stereotypies)	-->	F-04: Self-Injurious Behaviors	0.06	0.94			0.06		0.00	0.00	0.00			
61	F-23: Obsessive Compulsive Behaviors	<--	F-02: Isolated (Alone Preferred)	0.04	0.88			0.03	0.03	0.02	0.04				
62	F-17: Sensory & Object Preoccupation	-->	F-04: Self-Injurious Behaviors	0.04	0.96			0.04							
63	AGE	-->	F-16: Self-Confidence (SCI)	0.04	0.91	0.00		0.03		0.04		0.02			
64	AGE	-->	F-02: Isolated (Alone Preferred)	0.04	0.90	0.00		0.03		0.04		0.02			0.01
65	F-02: Isolated (Alone Preferred)	<--	F-08: Repetitive Speech (Perseverative Vocal Overflow)	0.04	0.93			0.00	0.04	0.00	0.02				0.01
66	F-16: Self-Confidence (SCI)	<--	F-08: Repetitive Speech (Perseverative Vocal Overflow)	0.04	0.95			0.00	0.01	0.00	0.04				
67	F-15: Socioemotional Awareness (Responsive/Expressive)	<--	F-17: Sensory & Object Preoccupation	0.03	0.94			0.00	0.02	0.00	0.03	0.00	0.00	0.00	
68	F-10: Staring (into Space; Preoccupied)	<--	F-15: Socioemotional Awareness (Responsive/Expressive)	0.03	0.96			0.00	0.03		0.01				
69	F-07: Motor Overflow (Excessive Impulsive Activity)	<-o	VIQ	0.02	0.97				0.01		0.00		0.02		
70	F-14: Body/Head Movements (Repetitive Rocking/Turning)	<--	VIQ	0.02	0.96		0.02				0.00		0.00		0.01
71	AGE	-->	F-01: Oppositional (Outburst Behaviors)	0.02	0.98	0.01		0.02							0.00
72	F-15: Socioemotional Awareness (Responsive/Expressive)	<--	F-05: Inflexible (Insistent Behaviors)	0.02	0.97			0.01	0.01	0.00	0.02				0.00
73	F-05: Inflexible (Insistent Behaviors)	<--	F-08: Repetitive Speech (Perseverative Vocal Overflow)	0.01	0.98				0.01		0.01				
74	F-14: Body/Head Movements (Repetitive Rocking/Turning)	-->	F-23: Obsessive Compulsive Behaviors	0.01	0.96			0.01	0.01	0.01	0.01		0.01		
75	F-16: Self-Confidence (SCI)	<--	F-07: Motor Overflow (Excessive Impulsive Activity)	0.01	0.99						0.01				
76	F-23: Obsessive Compulsive Behaviors	<-o	VIQ	0.01	0.99								0.01		
77	F-15: Socioemotional Awareness (Responsive/Expressive)	-->	F-23: Obsessive Compulsive Behaviors	0.01	0.99			0.01		0.00	0.00				
78	F-16: Self-Confidence (SCI)	-->	F-04: Self-Injurious Behaviors	0.01	0.99			0.00	0.00	0.01	0.00				
79	F-06: Social Atypicalities (Awkward, Odd Responses)	<-o	VIQ	0.01	0.99						0.00		0.01		
80	AGE	-->	F-05: Inflexible (Insistent Behaviors)	0.01	0.99			0.01		0.00		0.00			
81	F-08: Repetitive Speech (Perseverative Vocal Overflow)	<-o	VIQ	0.01	0.99						0.00		0.01		
82	F-16: Self-Confidence (SCI)	-->	F-06: Social Atypicalities (Awkward, Odd Responses)	0.01	0.99				0.00	0.01	0.00				0.00
83	F-04: Self-Injurious Behaviors	<--	F-06: Social Atypicalities (Awkward, Odd Responses)	0.01	0.99				0.01		0.00				
84	F-10: Staring (into Space; Preoccupied)	-->	F-01: Oppositional (Outburst Behaviors)	0.01	0.99			0.01	0.00	0.00	0.00				
85	F-17: Sensory & Object Preoccupation	<--	F-02: Isolated (Alone Preferred)	0.01	0.99				0.00		0.01				0.00
86	F-01: Oppositional (Outburst Behaviors)	<--	F-02: Isolated (Alone Preferred)	0.00	1.00				0.00		0.00				
87	F-14: Body/Head Movements (Repetitive Rocking/Turning)	-->	F-02: Isolated (Alone Preferred)	0.00	1.00			0.00		0.00					
88	F-15: Socioemotional Awareness (Responsive/Expressive)	<--	F-07: Motor Overflow (Excessive Impulsive Activity)	0.00	1.00			0.00		0.00	0.00				
89	F-16: Self-Confidence (SCI)	-->	F-05: Inflexible (Insistent Behaviors)	0.00	1.00					0.00					
90	F-01: Oppositional (Outburst Behaviors)	<-o	VIQ	0.00	1.00								0.00		
91	F-04: Self-Injurious Behaviors	<--	F-08: Repetitive Speech (Perseverative Vocal Overflow)	0.00	1.00				0.00		0.00				
92	F-15: Socioemotional Awareness (Responsive/Expressive)	<--	F-03: Hand/Body Movements (Recurring Mannerisms, Stereotypies)	0.00	1.00				0.00		0.00				
93	F-16: Self-Confidence (SCI)	<--	VIQ	0.00	0.99		0.00						0.00		
94	F-23: Obsessive Compulsive Behaviors	-->	F-04: Self-Injurious Behaviors	0.00	1.00			0.00		0.00					
95	F-08: Repetitive Speech (Perseverative Vocal Overflow)	<-o	NVIQ	0.00	1.00								0.00		
96	F-12: Socioemotional Unresponsiveness	-->	F-23: Obsessive Compulsive Behaviors	0.00	1.00			0.00		0.00					
97	F-16: Self-Confidence (SCI)	-->	F-01: Oppositional (Outburst Behaviors)	0.00	1.00					0.00					
98	F-17: Sensory & Object Preoccupation	<--	F-05: Inflexible (Insistent Behaviors)	0.00	1.00						0.00				
99	F-23: Obsessive Compulsive Behaviors	<--	F-07: Motor Overflow (Excessive Impulsive Activity)	0.00	1.00			0.00			0.00				
