# Supplementary material for: Integrating causal discovery and clinically-relevant insights to explore directional relationships between autistic features, sex at birth, and cognitive abilities
Source: Psychol Med. 2025 Mar 18;55:e89. doi: 10.1017/S0033291725000571 (PMC12080652; doi:10.1017/S0033291725000571)
Supplement: Tseng et al. supplementary material 3 — Tseng et al. supplementary material [file S0033291725000571sup003.docx]

| Term | Definition |
| --- | --- |
| Causal Discovery Analysis (CDA) | A process utilized to detect the causal relationship between variables |
| Causal Structure (Qualitative Causal Relationship) | A set of relationships between variables that defines which variables cause which variables |
| Conditional Independence Test | A statistical procedure that determines if two variables’ relationship is independent of the impact of a third variable |
| Eigenvalues | A scaler value that transforms a non-zero vector known as an eigenvector |
| Exploratory Factor Analysis (EFA) | A statistical technique that reduces data by identifying the number of contributing items and defines the structure of observed data |
| Greedy Fast Causal Inference (GFCI) | A 2-step process to make causal inferences – (Step 1) searches the space to establish potential causal relationships and (Step 2) an iterative refinement of the models, pruning false relationships |
| Latent Variables | An unobservable or unmeasured variable which impacts an observable measured variable |
| Maximum Likelihood Factor Extraction | An extraction method that returns the estimates most likely to have produced the observed data |
| Monte Carlo Permutation Analysis (parallel analysis) | A permutation test used when there are more possible orderings of the data than can be analyzed |
| Partial Ancestral Graphs (PAGs) | A graphical representation of causal relationships that included uncertainty, as the relationships between the nodes, the edges, are not fully specified |
| Structural Equation Modeling (SEM) | A set of statistical techniques that depict causal relationships between variables (observable and latent) |
